# A review of acupoint localization based on deep learning

**DOI:** 10.1186/s13020-025-01173-3

**Published:** 2025-07-22

**Authors:** Jiahao Li, Zhennan Fei, Yingjiang Xie, Da Deng, Xingcheng Ming, Fu Niu

**Affiliations:** Academy of Systems Engineering of Academy of Military Science of Chinese PLA, Beijing, China

**Keywords:** Acupoints localization, Deep learning, Keypoint detection, Survey

## Abstract

The development of deep learning has brought unprecedented opportunities for automatic acupoint localization, surmounting many limitations of traditional methods and machine learning, and significantly propelling the modernization of Traditional Chinese Medicine (TCM). We comprehensively review and analyze relevant research in this field in recent years, and examine the principles, classifications, commonly used datasets, evaluation metrics and application fields of acupoint localization algorithms based on deep learning. We categorize them by body part, algorithm architecture, localization strategy, and image modality, and summarize their characteristics, pros and cons, and suitable application scenarios. Then we sieve out representative datasets of high value and wide application, and detail some key evaluation metrics for better assessment. Finally, we sum up the application status of current automatic acupoint localization technology in various fields, hoping to offer practical reference and guidance for future research and practice.

## Introduction

In the theoretical framework of Traditional Chinese Medicine (TCM), acupoints are special locations on the human body that regulate the flow of vital energy (Qi) and blood through the meridians, and they are essential for applying therapeutic techniques such as acupuncture, moxibustion and massage [1]. By stimulating acupoints, one can achieve the goal of strengthening the body’s resistance and eliminating pathogenic factors, thereby treating diseases and promoting health and preventing illness [2]. For instance, in treating chronic conditions like rheumatoid arthritis, acupuncturists select specific acupoints according to their therapeutic properties. Primary points such as Zusanli (ST36), Guanyuan (CV4) and Shenshu (BL23) are commonly combined with supplementary points including Jiexi (ST41), Yangchi (TE4) and Baxie (EX-UE9). The warm-needle moxibustion technique is then applied to warm the meridians, enhance Qi-blood circulation and alleviate pain [3]. In terms of health preservation, TCM theory posits that insomnia stems from yin-yang imbalance and heart-kidney disharmony. Regular massage of acupoints like Yongquan (KI1) is thought to nourish kidney essence, boost vitality and improve sleep quality [4].

In modern medical research, the potential value of TCM acupoints should not be underestimated. Studies demonstrate that acupuncture stimulation can activate stem cell systems, trigger neurotransmitter and hormone release, induce immune responses and elicit characteristic physiological reactions including soreness, numbness, distension and pain [5]. Through systematic literature reviews combined with anatomical analyses, recent investigations have established significant correlations between facial acupoints and trigeminal nerve distribution. These findings provide scientific foundations for standardizing acupuncture protocols in treating related disorders [6]. In addition, some electrical properties of acupoints are different from those of the surrounding tissues, which provides a direction for studying the functions of acupoints and the assessment of related diseases from an electrical perspective [7].

However, the regulatory effects induced by acupoint stimulation exhibit high specificity and targeting precision [8]. Accurate acupoint localization is essential for delivering precise stimulation to corresponding meridians and visceral reflex zones. Traditional localization methods (e.g., proportional bone measurement, surface landmark identification, finger-cun measurement and simplified acupoint selection) present some limitations [9]. These techniques primarily depend on surface landmarks of the body (e.g., hairlines, bony prominences and muscular contours) combined with the measurement method using proportional units of the body. However, the anatomical structure of the human body varies from individual to individual and can also change over time due to postnatal factors. Finger-cun measurement results obtained from different fingers are highly variable and have low correlation. The bone measurements in different body parts are inconsistent, and factors such as individual body size, limb proportions, fatness or thinness, height and differences in the actions used for simple acupoint location all add to the complexity and uncertainty of locating acupoints. [10]. Consequently, these methods demand substantial practitioner expertise, often resulting in significant inter-operator variability.

In the context of medical-engineering integration, artificial intelligence (AI) has achieved groundbreaking progress across domains such as medical imaging diagnostics [11–13], disease prognostication [14–16] and pharmaceutical development [17–19]. As a subset of AI, machine learning enables computational systems to extract data-driven patterns for predictive modeling and decision-making, encompassing supervised [20, 21], unsupervised [22, 23], semi-supervised [24, 25] and reinforcement learning [26, 27] paradigms. Conventional machine learning, however, remains constrained by manual feature engineering when processing complex datasets. In contrast, deep learning (DL) architectures employing multilayer neural networks autonomously extract latent features from big data, primarily through convolutional neural networks (CNNs) [28], recurrent neural networks (RNNs) [29], autoencoders (AEs) [30], generative adversarial networks (GANs) [31], U-Nets [32], residual networks (ResNets) [33] and Transformers [34] models.

The application of deep learning in the medical field has advanced the development of acupoint localization. Specifically, DL-driven systems can extract acupoint morphological signatures and spatial patterns, integrating with TCM principles to achieve stable, high-precision localization. This technological advancement enables multifaceted applications: clinical acupuncture assistance through real-time localization guidance; self-care applications via smartphone-based massage zone mapping; educational standardization tools for accelerated acupoint mastery; diagnostic evaluation using thermographic acupoint temperature extraction; and robotic acupuncture/moxibustion systems requiring precise surface positions and depth information.

To systematically review the research progress of deep learning in acupoint localization, we established a scientifically rigorous literature screening framework and methodology. During the literature screening phase, multi-dimensional inclusion criteria were first defined: the research topic must focus on specific applications of deep learning algorithms (such as convolutional neural networks, generative adversarial networks, and Transformers) in human acupoint localization, while demonstrating the integration mechanism between TCM theory (e.g., anatomical characteristics of acupoints, meridian pathways and surface anatomical landmarks) and intelligent algorithms; methodologically, studies must outline a complete technical implementation pathway (from dataset construction and model development to localization validation) and provide objective quantitative or comparative qualitative performance evaluation metrics (e.g., localization accuracy, repeatability error, consistency validation, inference speed, model size, and computational complexity); the research type is limited to original articles or high-quality reviews published in the last five years (2020–2024) that include analysis of multimodal data (e.g., depth images, infrared thermography, ultrasound imaging); and the sources cover journals, conferences, and dissertations across biomedical engineering, computer science & artificial intelligence, TCM, medical robotics, and signal processing domains.

Based on these criteria, a hierarchical refinement strategy was implemented for literature retrieval. Initial screening utilized Chinese and English databases: CNKI, VIP Information, Google Scholar, Springer, Web of Science, IEEE Xplore, ACM Digital Library, PubMed, ResearchGate and arXiv, with the following combined keywords: (acupoint OR “acupuncture point” OR meridian) AND (localization OR detection OR identification), (deep learning OR deep neural network) AND “TCM robot”, and “massage robot” AND (deep learning OR “intelligent control”). References cited by relevant papers were also traced to expand source material. During the preliminary screening phase, articles were filtered by title and abstract to exclude non-acupoint localization studies and animal research, retaining approximately 180 publications aligned with the core themes. Subsequent full-text review identified studies explicitly employing deep learning methods, ultimately incorporating over 80 core publications. All selected literature underwent systematic deduplication using Zotero software. Key information–including body regions, algorithm architectures, localization strategies, imaging modalities and specific applications–was annotated via the software’s label management system.

**Fig. 1:**
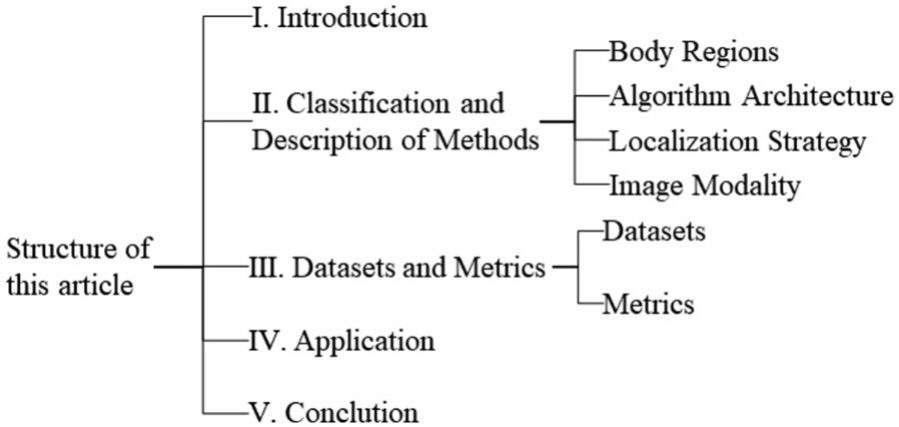
Thesis structure organization

This paper systematically reviews DL-based acupoint localization through five key aspects (Fig. 1):

1. Research background and significance;
2. Methods classification and analysis;
3. Datasets and evaluation metrics;
4. Application scenarios;
5. Conclusion.

## Classification and analysis of acupoint localization methods

With the rapid development of deep learning, its application in acupoint localization has become increasingly diverse. This section categorizes and analyzes DL-based methods from multiple perspectives to provide a comprehensive overview of the research paths and technical strategies. First, we explore the unique methods used for acupoint localization in different body regions, highlighting their difference. Next, we discuss the principles, advantages, and limitations of various deep learning frameworks, offering a solid foundation for future improvements. And then, we analyze different localization strategies, including direct localization, indirect localization, coordinate regression, Gaussian heatmaps, top-down, and bottom-up approaches, clarifying their core concepts, applicable scenarios and performance. This provides researchers and practitioners with a reference for selecting the appropriate localization method. Finally, we introduce progress in automatic acupoint localization using other image modalities, an area with limited research but high potential value for various applications.

### Localization in different body regions

The distribution of acupoints varies in density across different body regions, with complex and diverse anatomical landmarks and tissue structures, posing varying challenges for acupoint localization. For example, facial acupoints are close to vital organs and easily obstructed by hair; limb acupoints are influenced by posture and angle; and the torso lacks prominent features due to its relatively flat surface. These differences place varying demands on deep learning models for feature extraction and localization. Therefore, it is necessary to develop deep learning algorithms tailored to each body part, utilizing key region features and suppressing interference from different tissue backgrounds to improve localization accuracy.

#### Facial acupoint localization

The face is a crucial and intricate part of the body, with the eyes, ears, nose, mouth, and eyebrows, along with a complex network of nerves and blood vessels. It is densely populated with acupoints closely related to health, with special significance in TCM. Facial acupoint localization faces challenges such as facial expression changes, appearance differences, and lighting variations. Therefore, researching its localization methods requires considering multiple factors to build an accurate and adaptable model.

To improve accuracy and real-time performance in TCM facial acupoint augmented reality (AR) systems, Zheng et al. [35] enhanced the multi-task cascaded convolutional networks (MTCNN) face detection and practical facial landmark detector (PFLD) models. They developed three localization strategies: direct localization for coincident points (98.4% accuracy); proportional interpolation between landmarks (97.2%); polar coordinate-inspired positioning from single landmarks (96.8%). These methods enabled successful localization of 49 acupoints across 24 categories. The resultant mobile AR system provides real-time virtual acupoint visualization and information retrieval, offering public-friendly clinical assistance.

Chen et al. [36] applied transfer learning to overcome limited annotated data in facial acupoint localization. By transferring low-level features from a WFLW-pretrained facial landmark detection network (based on ResNet50), they developed an acupoint localization model using 1040 expert-annotated frontal grayscale images covering nine key acupoints. After data augmentation and fine-tuning with a newly initialized fully connected (FC) layer, transfer learning reduced the normalized mean error (NME) by 46.8%, and WingLoss optimization further reduced it by 16.7%. The model achieved an average NME of 2.5%, with minor accuracy variations in specific acupoints not affecting overall robustness.

Huang et al. [37] proposed a novel method for facial palsy diagnosis and quantitative analysis based on TCM acupoint recognition. The method first detects and crops faces using a model integrated in the Dlib library. After preprocessing, the cropped images are fed into a ResNet model pretrained on the ImageNet dataset for acupoint localization. The model then outputs 12-dimensional data corresponding to six acupoints. Finally, the degree of facial nerve damage is quantitatively analyzed and graded by measuring the angles between the lines connecting acupoints and the perpendiculars to the facial midline symmetry. Experimental results showed that the model performed well in acupoint recognition, with a NME of 0.0054 and an average angular error reduced to 4.5 degrees. However, the grading accuracy remained low for patients with significant facial expression changes.

Auricular acupoints differ from body acupoints as irregular segmented areas rather than point-like/circular regions, thus requiring segmentation algorithms supplemented by keypoint detection for precise delineation. Gao et al. [38] proposed a cascaded method. First, Faster-RCNN was used to detect the ear and crop the region. Then, the proposed K-YOLACT method simultaneously segmented auricular anatomical parts and detected keypoints. Finally, combining segmentation results, keypoints, and expert knowledge, OpenCV was used for image post-processing to achieve smoother and more refined auricular acupoint segmentation. Their approach achieved automatic segmentation of 66 regions with 83.2% segmentation mean average precision (mAP) and 98.1% keypoint mAP, demonstrating superior efficiency despite limitations in handling hair/earrings.

The above four methods focus on the localization of acupoints in specific regions. Zhang et al. [39] developed the E-FaceAtlasAR system in 2022, aimed at enabling non-experts to easily locate facial and ear acupoints. Leveraging the Mediapipe framework, the system overlays acupoint regions onto the user’s face in real time. For facial acupoint detection, it uses a pre-trained model for face alignment and hair segmentation, identifying 69 acupoints based on the B-cun method and facial landmarks. For ear acupoint localization, the system applies a standard template with ear detection, keypoint identification, triangulation, deformation, and fusion. Experiments show a facial acupoint NME of 3.9 and a failure rate of 5.13%. The system performs robustly under various angles and occlusions, while the ear segmentation method is sensitive to angle but resilient to lighting and occlusion, with higher error rates in complex ear structures, as indicated by the Dice coefficient.

#### Limbs acupoint localization

The limbs, as key areas for movement perception, are densely packed with acupoints related to limb function and the circulation of qi and blood. They have thick muscles, are distant from internal organs, and are safe for acupuncture. As major junctions of meridians, stimulating them can precisely regulate qi and blood, making them ideal for self-care. However, acupoint localization on the limbs faces challenges due to changes with movement and significant individual differences in muscle and skeletal structure. Therefore, deep learning methods should be combined with traditional localization or proportional methods to address individual variations and improve robustness and generalization.

To address the limitations in automatic hand acupoint localization, Chen [40] categorized hand acupoints into four groups based on distribution and selection criteria, using the middle finger as the reference. For acupoints detectable via hand keypoints, the Mediapipe keypoint detection algorithm is combined with TCM knowledge. For acupoints requiring contour detection, edge detection is employed. For nail-based acupoint localization, YOLOv8 is trained for nail detection, followed by offset localization using the bounding box. For acupoints without distinct features, regression neural networks are used based on keypoint coordinates. To accommodate the hand's flexibility and complex structure, rotational and functional methods are also proposed. Experiments reveal average localization errors (AE) of 9.96 for the first three categories and 14.8 for the Hegu (LI4) acupoint in the fourth category, meeting clinical accuracy standards. While the method is highly accurate, further refinement is needed for handling complex backgrounds.

Although the hand acupoint classification method mentioned above is more accurate, it still has significant speed limitations. Wang et al. [41] addressed the issues of low accuracy in lightweight networks and deployment difficulties with high-precision models by proposing a hand acupoint localization method based on a lightweight and efficient channel attention (LECA) network. Using MobileNetV2 as the backbone, the network incorporates an efficient channel attention (ECA) module for improved feature extraction and uses the HuberLoss function to handle varying detection difficulties across acupoints. The method successfully localized 11 acupoints on the palm. Compared to several classic models, it achieved higher accuracy with fewer parameters (only 1.67 M). However, it has the drawback of localizing fewer acupoints.

Given the long training time and low repeatability in manual acupuncture, Chan et al. [42] proposed a robotic acupoint localization method combining deep learning with TCM anatomical measurement. The system uses SSD-MobileNetv1 to detect regions and combines bounding box with TCM anatomical measurement for grid-based acupoint localization. The method was tested on the forearm with a dataset of 278 images, localizing 5 acupoints. Results showed that the mean offset errors (MOE) were all less than 0.08, successfully achieving acupoint localization and robotic acupuncture simulation. While other forearm acupoints can be localized through grid segmentation, the method also suffers from a limited number of localized acupoints.

Wang [43] conducted a study that combines global and local approaches. By using human pose recognition algorithms to obtain human joint points, the study then identifies the target regions based on these joint points. For regular body parts such as the forearm, the study employs skin color segmentation, corner detection, and the bone measurement method to locate acupuncture points. For complex parts like the hand, it uses key point localization and transverse finger width method to calculate the acupuncture points, and estimates the depth of the points through coordinate system transformation. Additionally, the study integrates the Kalman filter and optical flow method to solve the problem of re-localizing acupuncture points after body movement. The experimental results show that the average accuracy rates for locating acupuncture points on the forearm and hand are 94.5% and 93.4%, respectively, and the depth estimation accuracy rate is 96.4%. The method is mature and highly accurate, and can also be applied to acupuncture point localization on the lateral back and face. However, the evaluation metrics are relatively limited.

#### Torso acupoint localization

The torso, as a key region supporting the body's organs, contains numerous acupoints closely linked to organ function. Among them, Mu points regulate organ functions directly, the Ren and Du meridians gather qi and blood, and the Back Shu points help balance yin and yang, playing a crucial role in maintaining health. However, compared to the more three-dimensional face and limbs, the torso is relatively flat and lacks prominent features like facial landmarks or joint points. It is also significantly influenced by factors like breathing, posture, and body position, leading to considerable variability. Additionally, the dense distribution of acupoints on the torso increases the complexity of localization tasks.

Zhao [44] categorized back acupoints into obvious and fuzzy ones based on visual features, and proposed feature-assisted and segmentation-based localization methods respectively. The former used an improved ResNet50 to identify acupoint regions, located feature points with the Canny edge detection and corner detection algorithms, and obtained acupoint pixel coordinates via the finger-cun method. The latter segmented the back area with the SK-UNet semantic segmentation network, and located acupoints according to the intersection of segmented regions and the finger-cun method. Experiment results showed that this method had an average coordinate localization error of 4.68 mm and an average robotic arm localization error of 4.89 mm, meeting rehabilitation treatment requirements with high practicality and accuracy.

Fu et al. [45] used OpenPose to detect 18 human body keypoints and conducted bone proportional measurements. They extracted the mid-sagittal line and divided the distance from the shoulder keypoints to this line into six equal parts (each defined as GD). Based on GD, they calculated acupoint locations: for example, the Dazhui (GV14) point is located 3GD from the head keypoint along the mid-sagittal line; the bilateral Jianjing (GB21) points are the midpoints between the GV14 and the shoulder keypoints; and Shenshu (BL23) points are determined using TCM methods, based on the position of the fourth lumbar vertebra spinous process and a distance of 2GD. The acupoint coordinates were then converted from 2 to 3D and output to a robotic arm for back acupoint localization. Experiments demonstrated that the algorithm is accurate and practical for back acupoint localization under various camera conditions.

Liu et al. [46] proposed a method for acupuncture point localization based on prior information and deep learning, which localized 23 acupoints on the back. They first constructed an improved Keypoint RCNN network and embedded a convolutional block attention module (CBAM) to perform the initial localization of the acupoints on the back. Subsequently, based on the rules of acupoint distribution summarized from TCM theory and acupuncture experience, a posterior midline localization method was designed. This method involved image preprocessing, contour fitting, and centroid localization to determine the posterior midline, which was then used to correct the acupoints with larger initial localization deviations and to extend the localization of other acupoints. Experimental validation demonstrated that the improved model achieved an acupoint localization accuracy of 90.12%.

Feng et al. [47] proposed an abdominal acupoint detection and localization method based on meridian direction. They first used MediaPipe to extract key human posture points and combined YOLOv8 to detect the nipple and belly button, determining the main meridian directions based on these reference points. Then, using bone measurement methods and the established meridian coordinates, they localized 35 acupoints using geometric topology. Evaluation showed a mAP of 97.9% at IoU (intersection over union) = 0.5, and 87.1% for mAP@0.5:0.95, with an average distance error (MDE) of less than 3 mm, making it suitable for smart moxibustion and massage robots.

#### Multi-region acupoint localization

Due to the significant differences in characteristics among various body regions and the distinct methods for calculating acupoint coordinates, a localization method that reflects regional features is required for cross-regional acupoint positioning. Additionally, since multiple body regions need to be captured, the resolution is relatively low, and higher accuracy is demanded from the model.

Hu et al. [48] proposed a real-time acupoint localization method targeting the neck, back, buttocks and leg regions. The method first detects the human body using Faster R-CNN and then employs a Symmetric Spatial Transformer Network (SSTN) to estimate the human pose and obtain the coordinates of key skeletal joints. Subsequently, based on the skeletal node positions and joint vectors, the two-dimensional coordinates of the acupoints are determined using the bone measurement method. These 2D coordinates are then transformed into 3D coordinates. Experiments demonstrated that this method achieves a frame rate of 14 frames per second (FPS) and exhibits good robustness against various clothing colors and body postures. The average localization error for four commonly used acupoints is 2.36 cm. Although this error is relatively large for acupuncture robots, it is sufficient for massage robots.

#### Summary

**Fig. 2:**
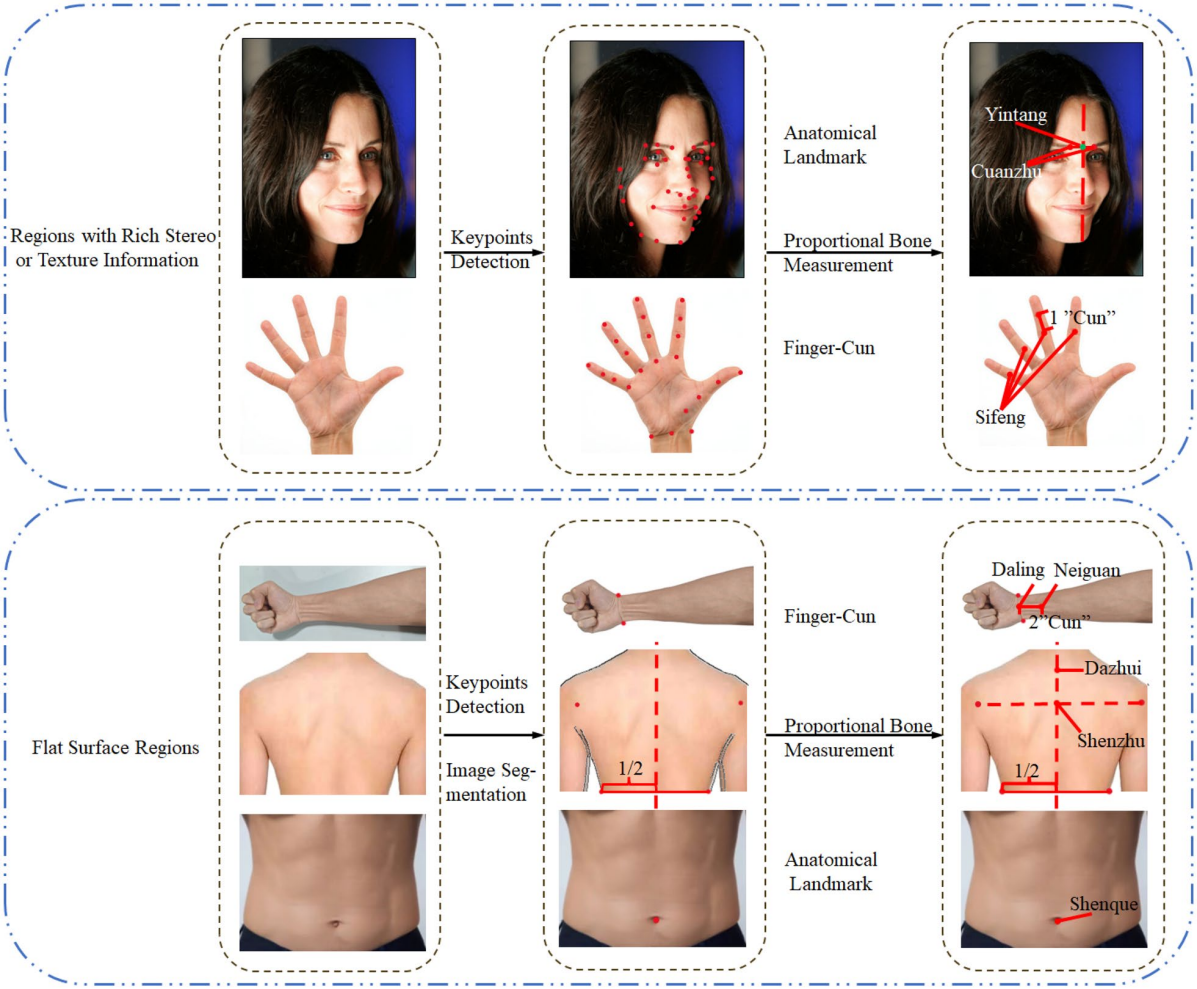
The general process for locating acupoints in different body regions

The human body’s anatomical regions have distinct structural features. Based on their distribution characteristics and surface traits, this paper categorizes them into three groups: head, limbs, and torso, as shown in Table 1. Figure 2 illustrates the general localization methods for each region. The face and hands, with rich surface features and easily recognizable landmarks, can be accurately localized using common keypoint detection algorithms combined with traditional methods. In contrast, the back, forearm, and chest/abdomen regions are relatively flat and have fewer distinctive features, so direct localization methods or special points combined with traditional measurements are typically used. While public keypoint detection datasets for the face and hands are abundant, the back has a dense distribution of acupoints, making it highly valuable for research in TCM meridians and acupuncture. Consequently, these three regions have been widely studied, generating a significant amount of related literature. However, research literature on automated acupoint localization across multiple body regions is relatively limited. The existing studies tend to focus on single or limited regions, and no method has yet been proposed that integrates the localization of acupoints on the face, back, and legs. Given the significant differences in characteristics among various body regions and the diverse localization requirements for each, it is imperative to develop a novel localization method that can accurately accommodate the unique features of each region.

**Table 1:** Summary of acupoint localization methods across different body regions

Body regions (number of acupoints)	Author (year)	Method	Evaluation metrics
Face			
Face (42)	Zheng et al. (2023) [35]	MTCNN face detection followed by PFLD for keypoint detection, then locating acupoints based on 3 positional relationships with keypoints	Direct (18 groups): mAcc = 98.4% Two-point Ratio (4 groups): mAcc = 97.2% Inch + Angle (2 groups) mAcc = 96.8%
Face (9)	Chen et al. (2021) [36]	Transfer learning with Resnet50 network	NME = 2.5
Face (6)	Huang et al. (2023) [37]	Dlib face detection + transfer learning based on pretrained ResNet	NME = 0.0054
Ear (66)	Gao et al. (2024) [38]	Faster-RCNN detection + K-YOLACT for keypoint detection and segmentation	mAP = 83.2%
Face (69) + Ear	Zhang et al. (2022) [39]	Alignment, segmentation, keypoint detection, B-cun (face), triangular mesh (ear)	NME = 3.9 (face)
Limbs			
Hand (23)	Chen (2023) [40]	Four methods: keypoint detection, edge detection, object detection, neural network fitting	ME = 9.96 (first 3 types) ME = 14.8 (4th Type)
Palm (11)	Wang et al. (2024) [41]	Lightweight keypoint detection network based on MobileNetV2	AP50 = 0.877; AR50 = 0.811
Forearm (5)	Chan et al. (2021) [42]	SSD-MobileNetv1 for arm detection + grid positioning method	GMOE = 0.06
Forearm (5) + Hand (9)	Wang (2021) [43]	Skin color segmentation, corner detection combined with bone measurement method (forearm), OpenPose keypoint detection + transverse finger width positioning method (hand)	mAcc = 94.5% (forearm) mAcc = 93.4% (hand)
Torso			
Back (37)	Zhao (2024) [44]	Back region recognition + keypoint detection Image segmentation + bone measurement	MDE = 4.68 mm
Back (7)	Fu et al. (2022) [45]	OpenPose keypoint detection + bone measurement	Almost indistinguishable from manual localization by doctors
Back (12)	Liu et al. (2023) [46]	Data augmentation with DCGAN, followed by improved KeypointRCNN model for direct localization	mAcc = 86.33%
Chest and abdomen (35)	Feng et al. (2024) [47]	MediaPipe for human pose keypoint detection + YOLOv8 for nipple and navel detection + bone measurement method (to determine meridians)	MDE < 3 mm
Multi-region			
Neck, back, buttocks and leg	Hu et al. (2021) [48]	Faster R-CNN human detection + SSTN keypoint detection + bone measurement	MDE = 2.36 mm

All evaluation metrics retain their original definitions and measurement units from the cited references

*mAcc* mean accuracy, *NME* normalized mean error, *ME* mean error, *AP* average precision, *AR* average recall rate, *GMOE* global mean offset error, *MDE* mean distance error

Overall, DL-based localization methods are numerous and mainly rely on techniques like GANs, object detection, and keypoint detection. Due to the unique shape of acupoints, the most commonly used methods are direct or indirect localization based on keypoint detection. However, despite advancements in keypoint detection and related technologies, automatic acupoint localization still faces several challenges that need improvement. First, datasets for acupoint localization are extremely scarce, and most are not publicly available, limiting data support and hindering progress in expansion and validation. Second, existing studies have issues with incomplete coverage and insufficient numbers of acupoints, which cannot meet the needs for in-depth research and practical applications. Third, some evaluation metrics are flawed, as they cannot comprehensively and accurately assess localization accuracy, and the lack of standardized criteria makes it difficult to objectively evaluate the effectiveness of various methods, impeding the optimization of research.

Although existing studies on acupoint localization methods have been able to adapt to factors such as angles, postures, and environmental changes, and have integrated TCM's cun measurement and surface landmarks, for acupoints that require precise localization dependent on specific postures (such as facial acupoints like Tinggong (SI19), Tinghui (GB2), and Ermen (TE21) with the mouth open, or limb acupoints like Hegu (LI4) and Quze (PC3) when joints are flexed), clear postural requirements for practitioners have not been specified. Although the targeted use of specific postures to localize individual or partial acupoints increases the workload of the dataset and the complexity of algorithm, this approach can effectively utilize the knowledge from traditional simple acupoint location methods, enhance the localization features, thereby improving the localization accuracy. In application scenarios with high requirements for localization accuracy of specific acupoints, such as VR-based teaching, this method not only improves the localization accuracy but also prompts the required auxiliary postures, thus optimizing the teaching quality.

### Localization using diverse algorithmic architectures

With the rapid development of deep learning technologies, many algorithms have emerged, each with unique advantages. In practical applications, we need to consider the specific situation and requirements to select the appropriate algorithm for optimization. For example, HRNet [50] excels at capturing high-resolution features, even with low-resolution input; GANs, with their strong generative ability, can enrich datasets while directly localizing acupoints; Transformers, utilizing self-attention mechanisms, can be more accurate and efficient in localization tasks; YOLOv8-Pose [56] meets the needs for real-time fast localization, making it suitable for quick-response scenarios; and the MediaPipe framework [59] and OpenPose library [61] provide rich models and tools for developers to deploy and develop applications. Furthermore, combining multiple models can effectively overcome the limitations of a single algorithm, improving accuracy and offering stronger support for intelligent applications in TCM. Below, we will explore the specific applications, advantages and disadvantages of these algorithms.

#### Acupoint localization based on GANs

GANs are a groundbreaking architecture in deep learning, consisting of a generator and a discriminator. The generator takes random noise as input and simulates the distribution of real data, generating realistic images. The discriminator, similar to a classifier, is responsible for distinguishing between real and generated images. Both networks continuously compete to optimize the generated data. GANs can uncover data features through unsupervised learning, generating data that closely approximates real samples, eliminating the need for manual feature extraction and enhancing modeling efficiency. They also expand datasets, helping train other models.

Gao [49] proposed a GAN-based localization method for acupoints near the eyes. First, a dataset of 600 images from 300 individuals, annotated by TCM experts with 18 acupoints near the eyes, was established. All images were resized to 512 × 512 via bilinear interpolation and aligned using Dlib facial landmarks, and an evaluation system consisting of outpoint rate (OAR) and Average coordinate error (ACE) was proposed for network training and objective assessment. To improve the efficiency, accuracy, and generalization of acupoint localization, the researcher introduced three GAN models. First, an improved Pix2pix network was used to propose a supervised learning acupoint estimation network, where the generator adopted a 16-layer U-Net structure and used the Adam optimizer, and only produced acupoint estimation maps. The discriminator was constrained with multiple precision conditions, leading to better localization speed and accuracy than the original network. Second, a supervised cyclic GAN was improved by adding a pyramid sub-attention module to the 8-layer U-Net generator to capture distant feature correlations, and a multi-scale attention module with several loss functions was integrated into the Patch discriminator. Although slower, this model achieved higher accuracy than the acupoint estimation network. Lastly, to address the lack of paired data, an unsupervised learning-based eye acupoint information separation and fusion network was proposed, with three generators designed to remove, extract, and fuse acupoint information with clean facial images. Experimental results showed that this network outperformed many traditional networks. The ACE for the 18 eye acupoints met clinical standards, although the Outpoint Rate for some acupoints was still low.

#### Acupoint localization based on HRNet

HRNet, introduced in 2019 by the University of Science and Technology of China and Microsoft Research Asia, is a groundbreaking approach to human pose estimation. It overcomes the limitations of traditional network architectures in handling multi-scale features by innovatively proposing a high-resolution network structure. Through parallel connections of multi-resolution branches and frequent information interaction, it enables complementary features from different resolutions, effectively improving pose estimation accuracy.

Zhang et al. [51] developed a human acupoint detection framework to address issues such as low detection efficiency, poor accuracy, and large errors in acupoint recognition across different body types in current intelligent moxibustion systems. They first applied the Faster R-CNN algorithm to detect the human body and then used HRNet for keypoint detection. By converting the “cun” value relationships based on TCM meridian theory, they computed acupoint coordinates using keypoints and acupoint data. The HRNet algorithm, with its unique parallel multi-resolution convolution structure, maintains high resolution throughout, avoiding the loss of feature information typical in traditional networks that downsample and then upsample. This enabled more accurate keypoint detection. The framework successfully detected 21 acupoints along the foot taiyang bladder meridian (mainly on the back), with errors not affecting the moxibustion treatment’s effectiveness, showing clear advantages in acupoint detection efficiency and accuracy compared to other methods.

Zhang et al. [52] improved the HRNet architecture by introducing a Resolution, Channel, and Spatial Attention Fusion (RCSAF) module before the head network. Using a dataset of 654 facial images (600 training/54 test samples resized to 256 × 256) annotated for 43 acupoints by licensed physicians, the RCSAF module leverages attention mechanisms (4-scale pyramid, 8 attention heads) to learn feature weights from different resolution paths and spatial locations, thereby effectively integrating features to produce more discriminative representations. The improved model was evaluated using Normalized Mean Error (NME) with normalization factor d set as inter-canthi distance (clinical standard for eye-related acupoints), Failure Rate (FR), and Area Under the Curve (AUC). When the model was trained with Adam optimizer and MSE heatmap loss, results indicated that the modified HRNet achieved the best performance, with an NME as low as 2.42%. Additionally, the study found that acupoints near facial landmarks (e.g., eyes, nose, and mouth) were localized more accurately, validating the effectiveness of using facial features to guide acupoint detection.

Seo et al. [53] evaluated HRNet and ResNet for acupoint localization on the hand and forearm, demonstrating HRNet's precision. Compared to ResNet, HRNet, with its high-resolution architecture, captured the fine details of acupoint positions more accurately. On a dataset with annotated arithmetic mean values by technicians, HRNet showed clear advantages. Even with low-resolution images (256 × 256), HRNet maintained a low average distance error. The HRNet-w48 model outperformed ResNet not only in various metrics but also in comparison with expert annotations, exhibiting superior localization accuracy.

#### Acupoint localization based on transformer

Transformer is a highly influential deep learning architecture that has gained significant attention in recent years. It consists of modules like multi-head attention, feedforward neural networks, normalization, and residual connections. Widely used in sequential tasks like natural language processing, it has also expanded to visual tasks and more. Transformer relies on the attention mechanism, which assigns attention weights based on the correlation between elements in the input sequence. Unlike RNNs and CNNs, it allows for direct parallel computation and excels at capturing global features. In handling long sequences, Transformer captures long-range dependencies via attention, avoiding gradient issues, and significantly reducing training time through parallel computation. Its strong versatility and scalability make it suitable for a variety of tasks.

Li et al. [54] proposed AIR-Net, a Transformer-based acupoint image registration network for automatic acupoint recognition and localization of 10 acupoints on the back of the hand. The network combines CNN and Transformer, using ResNet-50 for feature extraction and Transformer to learn the relationship between image pairs and acupoint positions. To ensure independent handling of acupoints and coordinate acquisition, the Transformer decoder removes the self-attention mechanism to prevent mutual interference, and the coordinates are then predicted through a fully connected layer. The self-attention mechanism in the Transformer encoder learns dependencies at different positions, compensating for CNN’s locality issues, thus capturing global features more effectively and improving localization accuracy. The experimental results showed an accuracy rate of over 90%, outperforming methods like T2RN and ResNet-101. It demonstrated good generalizability for images with different skin tones, orientations, and nails painted. However, the complexity of the Transformer structure makes the model large, and less robust in challenging conditions like cluttered backgrounds or poor lighting.

Yang et al. [55] proposed a keypoint detection network, AL-WFBS, for the localization of 84 acupoints on the human back with weak surface features. The architecture is based on the Detection Transformer (DETR) and mainly improves its Transformer module. A CNN is used to extract multi-scale features, which are then fused through a mixed encoder. The network employs an IOU-aware query selection mechanism to provide initial queries to the Transformer decoder. The decoder and auxiliary prediction head iteratively optimize the model, and the multilayer perceptron (MLP) and multi-head heatmap module perform acupoint classification and position regression to mitigate quantization errors. The method achieved an average precision error (AAPE) of 9.29 pixels (equivalent to < 1 cm physical distance) and PCK@0.05 (Percentage of Correct Keypoints at threshold 0.05) of 0.93, outperforming PVT/Swin-pose methods in accuracy. However, with an input resolution of 192 × 256 and GFLOPs of 151.8, the inference speed reached 13 FPS (72.7 ms per frame), barely meeting real-time requirements. This latency is attributed to the computational overhead of the hybrid encoder-decoder structure and the computational demands of heatmap regression. Future work should focus on optimizing lightweight deployment for clinical robotics applications.

#### Acupoint localization based on YOLOv8-Pose

YOLOv8-Pose is the most frequently used version in the YOLO series for keypoint detection. It inherits the high efficiency and accuracy of the YOLO family. The model uses a single-stage detection approach, combining object detection and keypoint detection tasks, simplifying the detection process and improving detection speed. This allows for rapid keypoint localization in real-time applications. Additionally, YOLOv8-Pose offers excellent scalability and adaptability. By improving modules such as backbone, neck, and head, it can flexibly handle keypoint detection tasks in various scales and complex backgrounds. It is widely used in fields like human pose estimation, facial expression analysis, and more.

Malekroodi et al. [57] applied YOLOv8-Pose to hand and arm acupoint localization, successfully identifying 5 acupoints using a data-driven method. As data-driven methods are direct localization and end-to-end, they built a dataset of 5997 annotated arm acupoint images from 194 participants. Various data augmentation techniques were applied to enrich the dataset, and the weights of the original YOLOv8-Pose model were fine-tuned using transfer learning, which enhanced the model's generalization and robustness. Under a 640 × 640 resolution, the average distance error for the 5 acupoints was less than 5 mm. They also developed an app that shows real-time acupoint locations, proving the method’s accuracy and real-time capability.

Yuan et al. [58] proposed the YOLOv8-ACU model for facial acupoint detection, which addresses the differences in facial acupoint features and human pose detection, as well as the high accuracy required for facial acupoint detection. The model mainly improves three parts: firstly, it adds the efficient channel attention (ECA) mechanism to the backbone network, enhancing the model's ability to extract global acupoint features. Secondly, it uses the Slim-neck module in the neck part, improving the detection of acupoints at different scales while reducing computational complexity. Finally, the loss function in the detection head is changed to generalized intersection over union GIoU to further improve detection accuracy. The experimental results show that YOLOv8-ACU outperforms models like YOLO-pose, YOLOv7-pose, and YOLOv8-pose in terms of accuracy, while also reducing model parameters and computational complexity. It demonstrates better performance and real-time capability in facial acupoint detection tasks.

#### Acupoint localization based on the MediaPipe framework

MediaPipe is an open-source multimedia processing framework developed by Google, which is highly versatile and adaptable for tasks involving images, videos, and audio. It offers developers a wealth of pre-trained models and reusable components, enabling rapid prototyping of applications. MediaPipe contains many efficient combination modules for applications such as object detection and facial landmark detection, and it also provides trackers and visualization tools for performance analysis and troubleshooting. Furthermore, it supports cross-platform deployment, allowing developers to test on desktop environments and then deploy to mobile devices, with easy component replacement for performance optimization.

Malekroodi et al. [57] not only proposed an acupoint localization method based on YOLOv8 but also developed an approach based on the MediaPipe framework, combining keypoint detection with proportional mapping for acupoint localization. They selected 20 commonly used facial acupoints and 18 hand acupoints. By utilizing MediaPipe’s FaceMesh and Handpipelines, they obtained keypoint coordinates for the face and hands. To address differences in acupoint visibility and localization accuracy from different poses or perspectives, they applied different indirect localization methods. For the hands, they divided the views into front, inner, outer, and rear, while for the face, they used central, left, and right perspectives. Using the detected keypoints as the foundation, they calculated acupoint positions based on traditional acupuncture literature's dimensional measurements. The final predicted acupoint average error was 5.58 pixels, approximately 0.3 cm, and the results could be displayed in real-time using an app.

Wei et al. [60] developed an AR interactive system for acupoint analysis, which provides users with an intuitive display of acupoint positions, helps them learn and understand acupoint knowledge, and offers diagnostic support through large language models and online doctors. The system leveraged the edge computing capabilities of the Jetson Nano J1010 development board, combined with a custom hand recognition model and MediaPipe technology to achieve acupoint localization and interaction. Similar to Malekroodi et al., the system relied on MediaPipe’s provision of 21 hand keypoints and 468 facial keypoints in 3D. Using a custom acupoint mapping algorithm, the system calculated acupoint positions based on these keypoints’ coordinates, relative distances, and mathematical functions like cross products and dot products. This allowed for accurate acupoint display on AR devices, with scaling based on distance. In situations where the hand rotated or stretched, the acupoint display accuracy reached 95.7%, providing users with an intuitive visualization of acupoint locations and assisting them in learning and understanding acupoint knowledge.

#### Acupoint localization based on the OpenPose library

OpenPose is an open-source real-time multi-person 2D pose detection library developed by the University of California, Berkeley (CMU). Compared to the MediaPipe framework, OpenPose uses non-parametric Part Affinity Fields (PAFs) to learn the associations between body parts and individuals in an image. This allows for more accurate multi-person pose detection in complex scenes, handling issues such as occlusion and body intersections. However, OpenPose tends to be slower in processing compared to other frameworks.

To address the needs of TCM massage, Lee et al. [62] developed a hand acupoint massage guidance method based on OpenPose keypoint detection and the proportional method. They also developed a corresponding app that can indicate acupoints and massage techniques based on the user’s condition. Using the OpenPose library, they first identified 20 keypoints on the back of the hand, then calculated acupoint positions based on the relative position of these keypoints according to TCM theory. While there was some error, it remained within an acceptable range considering the scope of the acupoint massage. The app, deployable on mobile phones, provides convenience for patients who can use hand acupoint massage to alleviate pain or treat diseases, offering personalized guidance for acupoint massage.

Wang [63] addressed the problem of automatic acupoint localization for intelligent moxibustion by designing a hand and arm acupoint detection method based on Lightweight-OpenPose and bone measurement methods. Lightweight-OpenPose is a lightweight model derived from OpenPose, with 85% fewer parameters but comparable performance. The researcher chose the arm as the measurement region for bone dimension and first segmented the arm area by converting the color space. Then, the minimum-area bounding box was used to obtain and correct the arm’s offset angle. Using hand width and gradient features, along with corner detection, the wrist and elbow crease positions were determined, with the distance set at 12 cun. The Lightweight-OpenPose model was used to locate baseline acupoints on the hand, and bone measurement methods were applied to calculate the coordinates of target acupoints. Ultimately, 6 acupoints were localized with an average accuracy of 95.8%, and the image processing speed was approximately 27 FPS, satisfying real-time requirements.

#### Multi-algorithm integration for acupoint localization

Different algorithms excel in their respective domains: YOLO is renowned for object detection, HRNet is proficient in keypoint detection, and U-Net excels in image segmentation. Leveraging these strengths, researchers have developed multi-stage integrated approaches to achieve more accurate acupoint localization. Typically, these methods first use object detection or image segmentation to obtain local images of interest, followed by keypoint detection on these local images. Finally, based on the detected key points, the bone measurement or finger cun measurement is used to precisely locate acupoints.

Yu et al. [64] proposed a hand acupoint localization method that combines YOLOv3 object detection and MediaPipe keypoint detection technology, specifically for difficulties in Mongolian acupuncture localization and its inheritance. They successfully localized 5 hand acupoints. The procedure involved the following steps: first, the YOLOv3 algorithm was used to identify the regions of the palm and back of the hand; then, the MediaPipe framework was employed to obtain the coordinates of 21 keypoints on the hand; next, the average method and polynomial fitting method were used to determine the distance and angular relationships between the hand keypoints and acupoints; finally, the specific coordinates of the acupoints were computed based on these relationships. Experimental results showed that the errors in the localization of the 5 acupoints were all within 1 cm, proving the efficiency and accuracy of this method.

Wang et al. [65] introduced a hand acupuncture point localization method based on a dual-attention mechanism (SE and CA) and a cascaded network model to improve the accuracy and robustness of acupuncture point localization. The method first used an improved SC-YOLOv5 model with dual attention mechanisms (SE and CA) to detect hand regions and generate bounding boxes. These bounding boxes were then input into a heatmap regression algorithm using HRNet as the backbone network to detect 21 hand keypoints. Finally, based on the TCM “MF-cun” (middle-finger width) measurement method, OpenCV was employed to calculate the coordinate relationships between keypoints and acupuncture points, achieving precise localization. Experimental results indicated that the method exhibited strong robustness in complex scenarios, with a mean offset error (MOE) of only 0.0269, a reduction of over 40% compared to other methods, while maintaining real-time performance (35 FPS).

#### Summary

As shown in Table 2, acupoint localization algorithms mainly cover three categories: generative adversarial networks (GAN), key point detection, and image registration. Among them, key point detection methods are the most widely applied, with a typical workflow involving the identification of key points first, followed by localization through traditional techniques such as the bone proportional measurement. In key point detection algorithms, HRNet stands out for its high precision in complex regions and low-resolution images, being widely used in multiple parts such as the face, back, and hands. Transformer can utilize global context to construct stable spatial relationships, making it particularly suitable for the back area with dense acupoints and also applicable to meridian construction. YOLOv8-Pose, MediaPipe, and OpenPose excel in fast computation and convenient deployment, fitting mobile device scenarios. Specifically, YOLOv8-Pose strikes a good balance between speed and accuracy, often used for distinct features like the face and hands. OpenPose specializes in full-body and hand key point detection, commonly seen in back and hand acupoint localization. Although MediaPipe can detect facial, hand, and full-body key points, its limited number of full-body key points and less-than-ideal positioning accuracy make it more applied to facial and hand acupoint localization. While GAN can be used for acupoint localization due to its image generation capability, its core value lies in data augmentation, effectively addressing the challenge of insufficient training data. Image registration-based methods, similar to template matching, show good adaptability to different postures and skin tones, but insufficient data training easily causes positioning deviations, leading to relatively fewer studies in this field. Since each algorithm has its own advantages, rational selection, combination, and optimization according to specific application requirements are particularly important.

**Table 2:** Summary of acupoint localization algorithms

Architecture	Author (Year)	Body part (number of acupoints)	Features	Evaluation metrics	
GAN	Gao (2024) [49]	Eye (18)	Proposed three GANs based on Pix2pix and CycleGAN	OAR	ACE
				70.67%	3.36
				75.11%	5.25
				71.33%	6.29
HRNet	Zhang et al. (2024) [51]	Back (21)	Calculated transverse finger width based on the distance between the right knee and right ankle	ME = 0.04	
	Zhang et al. (2023) [52]	Face (43)	HRNet with multiple attention mechanisms	NME = 2.42%	
	Seo et al. 2024 [53]	Dorsal hand (2) and Posterior forearm (3)	Demonstrated HRNet's advantage in low-resolution images	MPE = 4.81	
Transformer	Li et al. 2024 [54]	Dorsal hand (10)	A graph-based image registration network for acupoint localization	PCK-0.05 = 0.73 PCK-0.1 = 0.94	
	Yang et al. (2024) [55]	Back (21)	First use of Transformer-based keypoint detection for acupoint localization	PCK-0.05 = 0.89;	
YOLOv8-Pose	Malekroodi et al. (2024) [57]	Dorsal hand (2) and Posterior forearm (3)	Overcame dataset limitations using data augmentation and transfer learning	MDE < 5 mm	
	Yuan et al. (2024) [58]	Face (22)	Added ECA attention mechanism to YOLOv8-Pose and improved neck and loss function	mAP@0.5 = 99.5% mAP@0.5–0.95 = 80.7%	
MediaPipe	Malekroodi et al. (2024) [57]	Face (20) and Hand (18)	Calculated acupoint coordinates based on MediaPipe keypoints and proportion formulas	MDE = 3 mm	
	Wei et al. (2023) [60]	Face and Hand	Deployed MediaPipe-based acupoint localization algorithm on edge computing boards and integrated with large language models	Accuracy = 95.7%	
OpenPose	Lee et al. (2023) [62]	Dorsal hand (> 5)	Developed an OpenPose-based acupoint localization app for massage and health care	MPE = 8.04	
	Wang (2024) [63]	Palm (2) and Anterior forearm (4)	Lightweight-OpenPose combined with precise transverse finger width calculation	mAcc = 95.8%	
Multi-Algorithm	Yu et al. (2023) [64]	Palm (2) and Dorsal hand (3)	Using average and polynomial fitting methods to determine the distance and angle between hand keypoints and acupoints	MPE = 7.93	
	Wang et al.(2023) [65]	Palm (10) and Dorsal hand (10)	YOLOv5 with dual attention mechanism for hand detection + HRNet keypoint detection + MF-cun	MOE = 0.0269	

*OAR* Outpoint rate, *ACE* average coordinate error, *ME* mean error, *MPE* mean pixel error, *PCK* percentage of correct keypoints, *MDE* mean distance error, *mAP* mean average precision, *MDE* mean distance error, *mAcc* mean accuracy, *MOE* mean offset error

It is also important to note that keypoint detection-based algorithms include methods based on the Dlib library [66] and 3D Morphable Models (3DMM) [67]. However, in the field of acupoint localization, these two methods still rely heavily on machine learning components. Dlib excels in facial keypoint detection, while 3DMM offers strong representational power by simulating parameters such as shape, reflectance, and lighting, making it highly valuable for further research. Therefore, the deep learning components of these two technologies are worth exploring and expanding upon for more advanced acupoint localization methods.

### Localization with various strategies

To enhance DL-based acupoint localization accuracy, researchers have proposed various approaches with distinct strategies, each emphasizing different aspects of localization precision, real-time performance, and robustness. This paper categorizes these methods into six types based on three criteria: whether reference points are used (direct vs. indirect methods), regression formats (coordinate regression vs. Gaussian heatmap-based methods), and algorithm structures (top-down vs. bottom-up approaches). Analyzing these strategies provides better understanding of their principles and applicable scenarios, facilitating optimal method selection.

#### Acupoint localization based on direct method

The direct localization method leverages the powerful feature extraction and pattern recognition capabilities of deep learning, enabling acupoint localization directly from images without complex intermediate processes. The accuracy of this method primarily depends on three key factors: firstly, the expertise of the acupoint annotators; secondly, the diversity of the acupoint dataset; and lastly, the learning capability of the model. Currently, the model’s learning ability largely follows the scaling laws, meaning that within the limited scope of acupoint localization models, the quality and diversity of the data are especially crucial.

Compared to indirect methods, the direct localization method has the advantage of being simple, direct, and efficient, making it suitable for fast and automated acupoint detection tasks. Moreover, the acupoints in this method are independent of each other, avoiding the cascading failure phenomenon that can occur when errors in locating certain points lead to overall failures.

Wei et al. [68] adopted the Faster R-CNN model and successfully localized 13 common acupoints on the palm and 19 on the back of the hand using a self-built hand dataset. They first collected 2000 hand images, extracted key points using the MediaPipe pose estimation network, and then determined the positions of 32 acupoints in collaboration with teachers from a TCM university. They also applied data augmentation techniques to enrich the dataset. The model used ResNet-101 for feature extraction, and the Region Proposal Network (RPN) generated anchor boxes. A multi-task loss function was employed to ensure high localization precision. After several rounds of parameter tuning and training, the model achieved an mAP of 92% on the test set. They also developed an app for practical use.

Given that the effectiveness of current acupoint therapies heavily relies on the subjective judgment of doctors and medical resources are relatively scarce, Sun et al. [69] proposed an improved HRNet-based direct detection method for hand acupoints. This method can precisely locate 11 acupoints on the palm and 7 on the back of the hand. By introducing cascade hand detection, unbiased data processing, and a new loss function into the HRNet network, the accuracy of acupoint localization was significantly improved, with the maximum increase being 6.8%. With an error threshold of 0.06, the average error was about 3 mm, which is sufficient for acupoint massage and moxibustion treatments. The dataset used in this method was based on the 11KHands dataset, and after strict data cleaning, invalid images were removed, resulting in 3860 palm images and 4175 back-of-hand images. The images were resized, and acupoint labeling was based on commonly used clinical standards such as Chinese National Standards, expert observations, and visibility. Additionally, to enhance the model’s generalization ability, data augmentation techniques such as rotation, flipping, and brightness adjustment were applied.

#### Acupoint localization based on indirect method

Because the features on the human body’s surface are not always easily perceptible and certain environmental factors may interfere with feature recognition, acupoint localization accuracy can vary significantly. Unlike the direct method, which directly extracts surface features, the indirect method typically uses the reference point coordinates output by keypoint detection models or the corner points of bounding boxes obtained from object detection to assist with localization. This method helps improve localization accuracy by accurately identifying acupoints with distinct features as reference points, which are then used to determine the locations of other acupoints. Although this method may introduce some cumulative errors, the inherent coordinate relationships between acupoints generally result in higher accuracy compared to the direct method. Additionally, since only a few distinctly identifiable reference points need to be localized, the amount of annotated data required is relatively smaller. However, as the indirect method typically involves a two-stage or even three-stage localization process, the overall localization time is longer.

Su et al. [70] developed a multifunctional acupoint health care AR system, integrating three acupoint localization methods to address the challenges of acupoint localization and the lack of relevant knowledge among users. The three localization methods are point localization, point-to-point localization, and line intersection localization, which are mainly used for locating facial feature points through MLKit. This approach avoids the uncertainty introduced by angular factors in reference point, distance, and angle-based methods. Point localization is mainly used for acupoints located on key feature points; point-to-point localization applies to acupoints situated between two feature points that can be determined using proportional relationships; and line intersection localization is used for identifying acupoints near the intersection of two lines. Experimental results showed that the system achieved an overall NME of 3.376% when localizing 23 acupoints on the face. Although the error increased progressively with each method, the difference was not significant. Despite using a multi-stage localization process, the system’s speed was comparable to single-stage localization and could provide real-time display.

To improve the accuracy of hand acupoint localization, Zheng et al. [72] proposed a method combining hand reflex zones with keypoint detection. First, they used the DeeplabV3 network with an enhanced attention mechanism to segment hand images and identify the hand acupoint reflex zones (HAFZ). Then, they applied MediaPipe to detect the 21 keypoints of the hand and constructed the spatial topological relationships between these keypoints and the acupoints. Finally, the YOLOv8 algorithm was used to combine the constraints from the reflex zones and the topological relationships to further refine the acupoint locations. Ablation experiments showed that this approach significantly improved acupoint localization accuracy, especially for acupoints with less distinct features (such as the Yuji (LU10), Laogong (PC8), and Shaofu (HT8), where the improvement was more pronounced compared to using a single localization method.

#### Acupoint localization based on coordinate regression

The acupoint localization method based on coordinate regression is a strategy that directly predicts the coordinates of acupoints. This method first uses deep learning and other techniques to extract features from a large number of human body images, and then directly outputs the coordinates of the acupoints through fully connected layers or other network structures. The advantage of this method lies in its directness and efficiency, as it eliminates the need for complex image processing and coordinate calculation steps. This makes it more suitable for environments with limited computational resources, such as mobile devices. Additionally, since all acupoints share the same set of feature information, the relative positioning relationship between acupoints is maintained to some extent, allowing for reasonable localization results even when the input image quality is poor. Common keypoint detection algorithms based on coordinate regression include DeepPose [73], MTCNN [74], MobileNet [75], and PFLD [76].

Liu et al. [77] addressed the issue of slow automatic localization of facial acupoints by proposing a fast localization method based on an improved PFLD algorithm. They used MobileNetV3 as the backbone network and optimized the loss function. By adjusting the network parameters, they designed two versions of the network: FasterPFLD-S and FasterPFLD-L. In tests on the WFLW dataset, the FasterPFLD-S version achieved a 27.34% speed improvement over the original PFLD, with an inference speed of 5 ms per image and a NME of only 0.0571. Although the FasterPFLD-L version showed a smaller speed improvement (14.22% compared to FasterPFLD-S), it demonstrated higher accuracy and better performance in handling motion blur data.

Fei et al. [78] designed a vision-based waist and back meridian clearing robot, aimed at promoting the innovative development and inheritance of TCM. They used a 3D camera to capture RGB-D images of the human back, and acupoint locations were marked by TCM experts to construct the dataset. In their study, various deep learning models were trained and evaluated, including position regression-based ResNet, Gaussian heatmap-based UNet, and attention mechanism-based DMNet. Evaluation results showed that the UNet model based on Gaussian heatmaps achieved an average error of only 10 pixels, with high accuracy but poor robustness. The position regression-based ResNet model had slightly lower accuracy, but it exhibited strong robustness against external interferences such as lighting and clothing wrinkles and was able to maintain the structural correlation of acupoints. To ensure the stability and reliability of the robot’s application, the ResNet model was ultimately selected for deployment.

#### Acupoint localization based on gaussian heatmaps

The acupoint localization method based on Gaussian heatmaps employs an indirect approach by generating heatmaps to represent the locations of acupoints. Specifically, this method renders each acupoint as a Gaussian heatmap, with the network outputting multiple feature maps, each corresponding to one acupoint. The coordinates of the acupoints are then determined by identifying the maximum value point on the heatmap. The main advantage of this method is its high accuracy and the retention of spatial information. The heatmaps can capture a probabilistic distribution within a certain range, making this method generally more precise than coordinate regression. However, the computational cost is relatively high as it requires rendering Gaussian heatmaps and maintaining high-resolution feature maps, which increases the demand for computational resources and memory during both training and inference. As a result, the inference speed tends to be slower. Therefore, the Gaussian heatmap method is more suitable for applications that demand high localization accuracy. Common keypoint detection methods based on Gaussian heatmaps include Hourglass [79], OpenPose, and HRNet.

Chang et al. [80] proposed a hand acupoint localization method using ResNet152 as the backbone network in conjunction with the Gaussian heatmap approach for precise localization. They also introduced meridian assistance technology to help locate the acupoints. During training, they used a subset of images from the York University 11kHands dataset and employed various strategies to enhance the model’s performance. Notably, they introduced the SE (Squeeze-and-Excitation) attention mechanism, which effectively enhances the model’s ability to learn features from channels with high relevance, while reducing the weight of channels with less impact. This improved the concentration of the Gaussian heatmap results, significantly enhancing the recognition of features near complex acupoints.

Zhang et al. [81] proposed a facial acupoint detection model framework called FADbR, which integrates feature representation learning. The backbone of the model is an autoencoder network structure, and it predicts acupoint locations through the generation of heatmaps. The training process consists of two stages. First, in the self-supervised learning-based facial image reconstruction phase, the encoder compresses the input facial images into dense high-order representations, and the generator reconstructs the images based on these representations. The autoencoder is trained using four loss functions. In the second stage, during the facial acupoint detection phase, the autoencoder’s parameters are frozen, and convolutional layers are inserted into the inverse ResNet layer to extract features. These features are then mapped to heatmaps with 43 channels, and the model is trained using a heatmap prediction loss function. The acupoint coordinates are derived from the heatmap. Compared to other Gaussian heatmap-based models like SAN and HRNet, the FADbR model performs optimally across all metrics, with the majority of acupoint detection errors being under 10 pixels and the median error around 5 pixels. Furthermore, the model demonstrates stable performance even with small sample sizes.

#### Acupoint localization based on top-down approach

The top-down approach for acupoint localization first involves target detection of body parts in the image, followed by keypoint detection on those identified body parts. The advantage of this method lies in utilizing existing object detection technologies to locate each target, simplifying the subsequent keypoint detection process and improving the accuracy of keypoint localization for each target. However, since this method requires two processing steps, it tends to have higher computational complexity. Additionally, its accuracy depends heavily on the precision of object detection; if the target detection is inaccurate, errors may propagate into the subsequent acupoint localization. Common top-down keypoint detection algorithms include RMPE [82], CPN [83], and CPM [84].

Sun et al. [85] employed an improved CPM algorithm, using a top-down keypoint detection approach to directly localize the Quze (PC3) and Daling acupoints. They leveraged CPM’s multi-stage cascaded network structure. In the first stage, the input image is processed by a VGG-19 feature extractor, and then a 1 × 1 convolutional layer generates an initial confidence map reflecting the spatial distribution of the acupoints. In subsequent stages, the network's input includes both feature maps extracted from the original image and the confidence map generated in the previous stage, continually refining the accuracy of the confidence map. This results in precise acupoint localization. Additionally, to further optimize the localization, they constructed a real heatmap and calculated the L2 loss between the predicted heatmap and the real heatmap as the model's optimization target during training.

#### Acupoint localization based on bottom-up approach

The bottom-up acupoint localization algorithm first detects all the keypoints and then uses methods such as clustering to differentiate and associate these keypoints. This approach allows for the direct prediction of all the keypoints in an image without intermediate steps. The advantage of the bottom-up method is that the running time does not increase linearly with the number of targets, making it more suitable for real-time multi-person estimation. However, since keypoint detection is performed independently, it is more susceptible to noise, which may lead to inaccurate keypoint estimation. Common bottom-up keypoint detection algorithms include OpenPose and HigherHRNet [86].

Zhang et al. [87] proposed an improved OpenPose method for directly detecting 3 acupoints on the back. They optimized the OpenPose structure by retaining only the keypoint detection branch, reducing the number of parameters and speeding up the recognition process. They prevented gradient vanishing by using different convolution kernels at different stages and adopting the MSE loss function, which improved the training effectiveness of the model. After inputting a back image, the points with the highest response values on the heatmap were recognized as acupoints. The model was trained using the COCO2017 dataset and tested on their own dataset. The results showed that the probability of the three acupoints being covered by a 3.14 cm adhesive patch ranged from 73 to 76%, indicating significant room for improvement.

Zhang et al. [88] proposed a multi-task assisted acupoint search system using HigherHRNet as the backbone network to solve the acupoint localization problem. This system can locate 61 acupoints on the abdomen. The algorithm framework includes multi-task convolutional neural networks (CNNs), feature point localization, image coordinate alignment, and acupoint template overlay modules. The multi-task CNNs module uses HigherHRNet as the backbone, with branches to regress the coordinates of the Shenque (CV8) acupoint and segment the human body boundary. The feature point localization module determines 4 key points by finding the intersection of the CV8 acupoint and the human body boundary, and uses these to derive the coordinates of other acupoints. A module for image coordinate alignment and acupoint template overlay calculates the perspective transformation matrix from the obtained key points and maps the image coordinates to a standard space. Experimental results showed that the localization accuracy of 6 acupoints reached 97.9%. Although the accuracy is high, there are still issues, such as a limited number of validated acupoint categories and insufficiently detailed accuracy metrics.

#### Summary

As shown in Table 3, current acupoint localization strategies mainly focus on two aspects: accuracy and speed. On the one hand, methods such as direct localization, coordinate regression, and bottom-up approaches have been adopted to improve speed. On the other hand, indirect localization, Gaussian heatmap regression, and top-down methods have been developed to enhance localization accuracy. Given the importance of acupoint localization in medical applications and the significant functional differences among various acupoints, most studies have focused on improving localization accuracy, especially for applications like automatic acupuncture robots. Meanwhile, speed-oriented research targets real-time display applications, suitable for fields such as moxibustion, acupoint massage, and augmented reality-assisted localization. Among these, regression methods and architectures are not specific to certain categories of acupoints. However, the choice between direct and indirect localization methods significantly affects different acupoints: direct localization is typically used for acupoints with obvious features [such as Duiduan (CV25) acupoint at the midpoint of the upper lip tubercle], those with significant positional changes due to posture or muscle activity [such as Jiache (ST6) acupoint that varies notably with facial expressions], and those that cannot be indirectly localized using reference points [such as Daying (ST5) acupoint that requires tactile localization]. In contrast, indirect localization is suitable for acupoints with indistinct features but relatively fixed positions, which can be localized by relying on accurately positioned reference points [such as Yintang (EN-HN3) acupoint located between the eyebrows].

**Table 3:** Summary of acupoint localization strategies

Strategy	Author (year)	Body regions (number of acupoints)	Core method	Evaluation metrics
Direct	Wei et al. (2024) [68]	Palm (13) and dorsum (19)	Hand image acquisition using MediaPipe keypoint detection combined with acupoint annotation by TCM experts	mAP = 92%
	Sun et al. (2022) [69]	Palm (11) and dorsum (7)	Data cleaning and augmentation of public datasets, annotated according to Chinese national standards, verified by professional doctors	MDE = 3 mm
Indirect	Su et al. (2023) [70]	Face (23)	Three localization methods based on the relationship between acupoints and keypoints: point localization, inter-point localization, and intersection localization	NME = 3.376%
	Zheng (2024) [72]	Hand (16)	YOLOv8 fusion of acupoint reflex zone maps and acupoint maps indirectly obtained by MediaPipe	MDE = 3.91 mm
Coordinate Regression	Liu et al. (2023) [77]	Face (16)	Improved PFLD algorithm with MobileNetV3 as the backbone network and optimized loss function	MPE = 2.21
	Fei et al. (2023) [78]	Back	Comparison of Resnet with coordinate regression and Unet with Gaussian heatmap	Heatmap is accurate; Regression is robust
Gaussian Heatmap	Chang et al. (2024) [80]	Hand (11)	Introduced SE attention mechanism into ResNet for Gaussian heatmap regression	mAcc = 91.72%
	Zhang et al. (2023) [81]	Face (43)	Constructed FADbR framework with autoencoder as the backbone, higher precision than SAN and HRNet	NME≈1.9%
Top-Down	Sun et al. (2020) [85]	Quze and Dalin	Improved CPM algorithm with VGG-19 as the backbone network and optimized loss function	ADR = 76%
Bottom-Up	Zhang et al. (2024) [87]	Back (3)	Removed the branch for connecting keypoints in OpenPose to improve efficiency	ADR = 74.88%
	Zhang et al. (2024) [88]	Abdomen (61)	Multi-task convolutional neural network with HigherHRNet as the backbone	Accuracy = 97.9%

*mAP* mean average precision, *MDE* mean distance error, *NME* normalized mean error, *MPE* mean pixel error, *mAcc* mean accuracy, *ADR* average detection rate

It should be noted that, although most algorithms follow certain patterns regarding speed and accuracy, they still require verification using actual datasets for specific applications. For example, Kuang [104] pointed out in their research that compared to the Gaussian heatmap-based HRNet network, which has a larger parameter size and higher computational complexity, the coordinate regression-based PFLD network performed better in terms of inference speed, memory usage, and localization accuracy. Acupoint localization via image registration [89] can fully utilize acupoint structural information to reduce local deviations and achieve higher-precision localization. Most of the above-mentioned localization methods are data-driven, requiring large-scale datasets for ideal results. Conversely, transfer-learning-based acupoint localization [90] can effectively overcome the limitations of manual annotation and rule-based matching, as well as address accuracy issues.

### Localization across different imaging modalities

Currently, most acupoint localization methods are based on visible light images. However, to overcome the limitations of visible light information and achieve more accurate localization, researchers have started exploring other imaging technologies, such as depth cameras, thermal imaging, and medical imaging devices. These different image modalities reflect various aspects of the human body, and thus often have more specialized applications.

#### Acupoint localization based on depth images

In recent years, the rise of various TCM robots, such as acupuncture robots, moxibustion robots, and massage robots, has broken through the limitations of two-dimensional planes for automatic acupoint localization, enabling further determination of distances. The key to this advancement lies in the use of depth cameras, which can capture RGB-D images through either passive or active means and map coordinates to 3D space.

Masood [91] proposed an indirect 3D hand acupoint localization method for intelligent moxibustion robots to achieve more accurate positioning. This method integrates an RGB-CNN model based on VGGNet with a Depth-CNN model. By learning and merging features while continuously optimizing the fusion parameters, a multimodal fusion detection acupoint model was developed that directly outputs three-dimensional acupoint coordinates. Experimental results showed that this method could localize five sets of acupoints with an average localization error of less than 0.09, demonstrating its potential application value.

#### Acupoint localization based on infrared thermography

All objects emit infrared radiation, a property that allows thermal cameras to detect the temperature distribution on the surface of objects and convert it into visible images using long-wave infrared detection equipment. Acupoints, as areas where qi and blood converge, typically have a noticeable temperature difference compared to surrounding skin temperature [92]. Therefore, acupoint localization based on thermal imaging can, to some extent, leverage the temperature characteristics of acupoints, providing new approaches and methods for TCM treatment.

As early as 2012, Zhao et al. [93] localized multiple acupoints in facial thermal images using corner detection and edge detection methods, applying it to facial paralysis treatment. Other studies [94–98] have also employed corner detection, edge detection, model building, and machine learning methods to locate acupoints on thermal images. However, deep learning methods in this field are still relatively few and have significant room for development.

In response to the COVID-19 pandemic, where conventional non-contact infrared thermometers could not detect masked individuals and lacked sufficient accuracy, Xia [99] proposed an intelligent infrared thermometer based on Raspberry Pi, which can identify mask-wearing and detect the temperature of acupoints. Using the SSD algorithm, the device first detects mask-wearing conditions, and then uses the Dlib keypoint detection algorithm in combination with a temperature calibration algorithm to measure the temperature of the acupoints on the forehead, including Cuanzhu (BL2), Yintang (EN-HN3), and Yuyao (EN-HN4). Experimental results showed that the measurement error was 0.2 °C, demonstrating its high practical value.

#### Acupoint localization based on medical imaging

Acupoints hold an extremely important position in TCM. With the continuous advancement of medical imaging technologies, many researchers have started exploring the precise location of acupoints in various medical image modalities. By accurately locating the depth of acupoints beneath the skin tissue, it is possible to achieve more precise acupuncture treatments, while also providing strong support for the scientific study of acupoint theory. However, research on acupoint localization using deep learning in the context of medical imaging remains relatively limited.

Liu et al. [100] proposed a direct acupoint localization method based on an improved YOLOv7 model, specifically designed for locating the Baliao acupoints (BL31–BL34) in sacral CT images. In this method, the Ghost module replaced part of the ELAN modules in the backbone network, effectively reducing the computational complexity and number of parameters. Additionally, by incorporating the Channel Attention (CA) mechanism, the model was able to focus on both channel and spatial information, improving its detection performance for these acupoints. The study used a dataset constructed from 60 sacral CT (Computed Tomography) images of patients provided by a hospital in Suzhou. Experimental results showed that the modified model improved its mAP@0.5 from 88.5 to 93.1%, and its FPS increased from 53.4 to 78.9. The predicted values, compared to the actual values provided by the hospital, had errors within a safe range, indicating that this model could be applied to laser penetration therapy.

#### Summary

As shown in Table 4, multi-modal imaging has introduced new applications for acupoint localization. Among them, methods based on depth cameras can capture distance information, which in turn helps control the operation intensity of acupuncture or massage robots. Methods utilizing thermography can acquire temperature information from acupoints, which is of significant value for disease diagnosis and the assessment of treatment effects. Meanwhile, methods based on medical imaging technologies provide additional anatomical details, offering more precise positional references for medical device operations, playing a crucial role in clinical applications.

**Table 4:** Multimodal imaging approaches for acupoint localization

Imaging modality	Author (year)	Body regions (number of acupoints)	Application	Evaluation metrics
RGB-D Imaging	Masood [91] (2022)	Hand (15)	Moxibustion robot	ME < 0.09
Infrared Thermography	Xia [99] (2023)	Cuanzhu, Yintang, Yuyao	Smart non-contact infrared thermometer	TE = 0.2 °C
CT Imaging	Liu et al. [100] (2024)	Baliao (Eight Lumbar Points)	Laser penetration therapy	mAP@0.5 = 93.1%

*MDE* mean distance error, *TE* temperature error, *mAP* mean average precision

Currently, most research on acupoint localization using deep learning has focused on RGB images captured by visible light. However, studies on modalities like depth images, infrared thermal images, and medical images—which contain less feature information—are relatively scarce. Among these modalities, infrared thermal and medical imaging typically rely on single-modal localization, while depth images are often combined with visible light images to achieve more precise localization. Given the significant research value of multi-modal images in modern medicine, future research should explore how to extract more features from these images to enable even more accurate acupoint localization.

The progress of deep learning in the field of computer vision (CV) and its advantages in visual positioning have made image-based automatic acupoint localization the mainstream method. However, the localization of some acupoints relies on palpation in traditional methods (such as perceiving bone seams, depressions, or protrusions). To compensate for the limitations of single visual modality in such tactile information, a pressure sensor array can be introduced and applied to the massage robot system. By fusing image modality and pressure sensing data, a multimodal perception model is constructed to improve the accuracy and robustness of acupoint localization.

## Datasets and evaluation metrics

The accuracy of DL-based acupoint localization models is highly dependent on the quality and diversity of the datasets used. An ideal dataset should contain a wide variety of image samples, covering individuals of different ages, genders, body types, as well as diverse environments and postures. This diversity helps the model learn a broad range of features and enhances its generalization ability. Moreover, to ensure effective comparison between different models, standardized evaluation metrics should be used, such as the mean absolute error (MAE), root mean squared error (RMSE), and detection success rate. These metrics provide an objective measure of the model's localization accuracy and robustness, helping researchers select the most suitable model for practical applications.

### Acupoint localization datasets

In this paper, the datasets are categorized based on the localization strategy into direct localization datasets and indirect localization datasets. Direct localization datasets include the localization images and their corresponding acupoint coordinates, while indirect localization datasets are primarily used for keypoint detection to provide reference points for auxiliary localization. Based on the body regions covered by the direct localization datasets, they are classified into three categories: facial, limb, and torso datasets, with the goal of covering various parts of the body. Given the large number of indirect localization datasets, only some commonly used and important datasets are introduced.

#### Facial acupoint localization datasets

FAcupoint Dataset [101]: this dataset supports the research on the recognition of 24 types of facial acupoints (43 acupoints in total). It contains 654 grayscale face images with a resolution of 480 × 360 pixels in TIFF format. The dataset includes detailed information such as gender, age, identity, lighting conditions, posture, and facial expressions. The acupoint locations were annotated and reviewed by five licensed Chinese medicine practitioners, and the final ground truth was based on the average of their annotations.

Yuan et al. [58] established two facial acupoint detection datasets. Dataset I selected 608 front-facing images with minimal occlusion from the WIDER Face dataset, annotated by three senior acupuncturists following WHO standards for 22 facial acupoints across 11 categories. Dataset II collected 236 front-facing photos of Asian and Caucasian adults (1:1 gender ratio) from online sources and the SCUT-FBP5500 dataset, annotated identically for evaluating model generalization.

Chen et al. [36] constructed a dataset containing nine facial acupoints [bilateral Sizhukong (TE32) and Cuanzhu (BL2), Suliao (GV25), Duiduan (CV25), Chengjiang (CV24), and bilateral Dicang (ST4)] through collaboration with acupuncture experts. The dataset includes 1040 front-view grayscale images across different ages and genders.

Eye Acupoint Dataset: Gao [49] collected 600 images (512 × 512 resolution) of eye acupoints from 300 volunteers. Experienced acupuncturists with over 10 years of expertise annotated 18 eye-related acupoints. The dataset was expanded to 800 images for unsupervised learning, ensuring sufficient data for model training and evaluation.

For automated auricular acupoint localization, Sun et al. [102] developed the ear image dataset (EID) using custom handheld devices to capture 504 ear images (500 × 500 resolution) from 252 participants. Guided by TCM specialists, 91 auricular key points (31 primary and 60 secondary) were manually annotated following the GB/T13734-2008 standard [71] (National Standard of China), with high inter-rater reliability confirmed by intraclass correlation coefficient (ICC) analysis.

Gao et al. [38] created an auricular dataset comprising two components: (1) A human ear detection dataset with 1070 multi-angle ear images from Hangzhou Normal University, featuring varying hair occlusions; (2) An ear region segmentation and key point prediction dataset containing 200 randomly selected ear images from USTBHelloear, annotated with anatomical regions and key points.

#### Limb acupoint localization datasets

Sun et al. [69] developed a hand acupoint detection dataset based on the 11 K Hands dataset, which originally contains 11,076 hand images from 190 subjects aged 18–75 years. After curation, 3860 palm images and 4175 dorsum images were selected and uniformly resized to 1600 × 1200 resolution. Following national standards, clinical expertise, image visibility, and common clinical usage, 11 palm acupoints and 7 dorsal acupoints were annotated.

Seo et al. [53] created the PK dataset for localizing three arm and two hand acupoints. They collected arm images from 94 individuals, processed into 940 images (1488 × 837 resolution). Under medical expert supervision, four technical assistants annotated the data using the bone proportional measurement method. Intra-group and inter-group experiments revealed significant variability, mitigated by averaging coordinates across annotators.

Li et al. [54] utilized the 11 K Hands dataset for dorsal hand acupoint localization. TCM practitioners annotated 10 dorsal acupoints, with the distance between the first and second metacarpophalangeal joints standardized as one cun. To enhance network robustness and reduce annotation time, data augmentation (rotation, flipping, scaling) was applied. Image pairs were generated through random matching to expand the dataset.

Zheng et al. [72] selected 1000 left-hand palm images from the 11 K Hands dataset, annotating 16 distinct acupoint categories to support both point localization and reflex zone segmentation.

Wang et al. [41] constructed a palm acupoint dataset containing 1170 single-palm images, 11 acupoints were annotated, including Shixuan (EX-UE11) at fingertips, Sifeng (EX-UE10) at finger joints, Laogong (PC8), and Shaofu (HT8) on the palm.

For wrist and forearm acupoint localization: Cai et al. [103] built a dataset with 110 images from 56 volunteers, annotating Taiyuan (LU9), Daling (PC7), and Shenmen (HT7). Sun et al. [85] developed a 719-image dataset for Quze (PC3) and PC7 localization. Chan et al. [42] established a 278-image dataset targeting five arm acupoints.

#### Torso acupoint localization datasets

Kuang [104] collected 7010 exposed back images covering diverse postures, age groups, heights, and genders. Using the structural similarity index measure (SSIM) algorithm for deduplication, 6109 valid images were retained. Forty-three acupoints along the Bladder Meridian, Governor Vessel, Small Intestine Meridian, and extra points were localized via bone proportional measurement formulae.

Liu et al. [46] acquired back images through photography and web crawling, maintaining a 1:1 ratio of muscular tension/relaxation states. The dataset was expanded to 4000 images (1500 × 2000 resolution) using data augmentation and DCGAN synthesis. Twelve clinically significant back acupoints and detection areas were annotated through combined bone measurement and empirical methods. Zhang et al. [87] similarly processed images through deblurring and filtering, with Shenzhu (GV12) and bilateral Naoshu (TE10) annotated by TCM university students.

Ji [106] developed two datasets for monocular camera-based 3D acupoint localization: the monocular 3D human reconstruction dataset, which was generated using 6000 images from skinned multi-person linear (SMPL) model projections; and the point cloud acupoint localization dataset, which was created by generating 10,100 synthetic human models via the smplx (Python package) with randomized shape parameters and neutral poses. Acupoints including Dazhui (GV14), Dazhu (BL11), Fengmen (BL12), and Xinshu (BL15) were localized using finger-cun measurement for model training.

Yang et al. [55] recruited approximately 200 volunteers (healthy, non-scoliotic, varying BMI) to build an RGB-D image dataset with 84 back acupoints. Annotated by three licensed TCM practitioners following the National Standard GB/T 12346-2021 [105] (Nomenclature and Location of Acupoints).

Zhang et al. [88] established an abdominal acupoint dataset for abdominal acupuncture systems, containing 500 images annotated with Shenque (CV8) and abdominal boundaries.

#### Indirect acupoint localization datasets

WLFW Dataset (Wide-Range Face Web) [107]: this dataset focuses on facial keypoint detection and includes 10,000 images of faces. Each face has 98 manually annotated key points, with additional annotations for pose, expression, illumination, makeup, occlusion, and blur.

 300 W Dataset [108]: this dataset is another important dataset in the field of facial keypoint detection. It is an amalgamation of multiple publicly available datasets and includes 300 indoor facial images and 300 outdoor images. Each image has 68annotated key points, capturing various poses, expressions, lighting conditions, and occlusion.

OneHand10K Dataset [109]: this dataset contains 11,703 RGB images of a single hand. Each image has 21 annotated key points on the hand, and the dataset also includes manual hand segmentation. It covers various gestures, interactions between the hand and objects, complex backgrounds, severe self-occlusion, and different lighting conditions.

The RHD dataset [110]contains both of hands, with a total of 41,258 training samples and 2728 test samples of RGB images, depth maps, segmentation masks, and the 21 key points of each hand, annotated with UV coordinates, XYZ coordinates, and visibility. This dataset can be utilized for hand acupoint localization, especially in 3D scenarios where depth information is essential.

COCO Keypoints 2017 Dataset [111]: this dataset is widely used for human pose estimation and contains over 200,000 images, with more than 250,000 human instances annotated. Each instance is marked with 17 key points, including the head, shoulders, elbows, wrists, hips, knees, and ankles, across various everyday scenes.

MPII Dataset [112]: this dataset is a key benchmark in human pose estimation, comprising approximately 22,000 images with over 40,000 annotated human instances labeled with 16 keypoints. Images are sourced from YouTube videos, covering 410 diverse daily activities and include corresponding activity labels. It features rich diversity, complex scenarios, and varied viewpoints, supporting both single-person and multi-person pose estimation.

#### Summary

As shown in Table 5, acupoint localization datasets are diverse, covering various body parts like the face, eyes, ears, hands, arms, back and abdomen. However, direct localization datasets are generally not publicly available and have limited data, while indirect localization datasets, though publicly accessible, lack precision and authoritative annotations.

**Table 5:** Datasets for acupoint localization

Category	Author (year)	Location (number of acupoints)	Number of images	Resolution	Annotation method	Public availability
Head	Zhang (2023) [101]	Face (43)	654	480 × 360	Annotated by 5 licensed physicians	Semi-public
	Yuan et al. (2024) [58]	Face (22)	Class I: 608 Class II: 236		Jointly annotated by 3 acupuncturists following WHO standards	Semi-public
	Gao (2024) [49]	Eye (18)	600	512 × 512	Annotated by acupuncturists with over 10 years of experience	Private
	Sun et al. (2022) [102]	Ear (91)	504	500 × 500	Four annotators trained by a professional physician, with reliability assessed using intraclass correlation coefficient	Private
	Gao (2024) [38]	Ear	ED^a^: 1070 ES^b^: 200		Annotated by researchers using Labelme tool	Private
Limbs	Sun et al. (2022) [69]	Palm (11) and Dorsum (7)	3860	1600 × 1200	Annotated based on national standards with some verified by physicians	Private
	Seo et al. (2024) [53]	Dorsal hand (2) and Posterior forearm (3)	940	1488 × 837	Annotated by four assistants under the guidance of medical experts, with intra- and inter-group consistency assessed	Private
	Li et al. (2024) [54]	Dorsal hand (10)	from 11 K dataset	1600 × 1200	Acupoints and transverse finger width annotated by TCM practitioners	Private
	Zheng et al. (2024) [72]	Left palm (16)	1000	640 × 640	Manually annotated by researchers	Private
	Wang et al. (2024) [41]	Palm (11)	1170		Manually annotated by researchers	Private
	Cai et al. (2022) [103]	Wrist (3)	110	576 × 768	Manually annotated by acupuncturists	Private
	Sun et al. (2020) [85]	Quze and Dalin	719		Manually annotated by researchers	Private
	Chan et al. (2021) [42]	Anterior forearm (5)	278		Manually annotated by researchers	Private
Torso	Kuang (2023) [104]	Back (43)	6109		Annotated using bone measurement method based on literature formulas, with SSIM algorithm for deduplication	Private
	Liu et al. (2022) [46]	Back (12)	4000	1500 × 2000	Annotated combining bone measurement method and experience-based method	Semi-public
	Ji (2024) [106]	Dazhui acupoint	RD ^c^: 6000; PC ^d^: 10,100		Manually annotated the Dazhui acupoint by researchers	Private
	Yang et al. (2024) [55]	Back (84)			RGB-D images of the back annotated by three TCM practitioners using Labelme	Private
	Zhang et al. (2024) [88]	Abdomen (6)	540		Training set: Manually annotated Shenque acupoint and body boundaries; Test set: 6 validation acupoints marked by professional doctors	Private
Indirect	WLFW (2018) [107]	Face (98)	10,000		Manually annotated by researchers	Public
	300 W (2013) [108]	Face (68)	3837		Semi-supervised annotation	Public
	OneHand10K (2018) [109]	Hand (21)	11,703	1600 × 1200	Manually annotated with cross-validation	Public
	RHD (2019) [110]	Hand (21)	43,986	320 × 320	Construct with models and animations and expand by introducing other datasets	Public
	COCO Keypoints (2017) [111]	Whole body (17)	> 200,000			Public
	MPII (2014) [112]	Whole body (16)	~ 22,000		Crowdsourced manual annotation	Public

^a^ED is ear detection dataset

^b^ES is ear segmentation dataset

^c^RD is monocular image-based 3D human reconstruction dataset

^d^PC is point cloud dataset for human acupoint localization

**Fig. 3:**
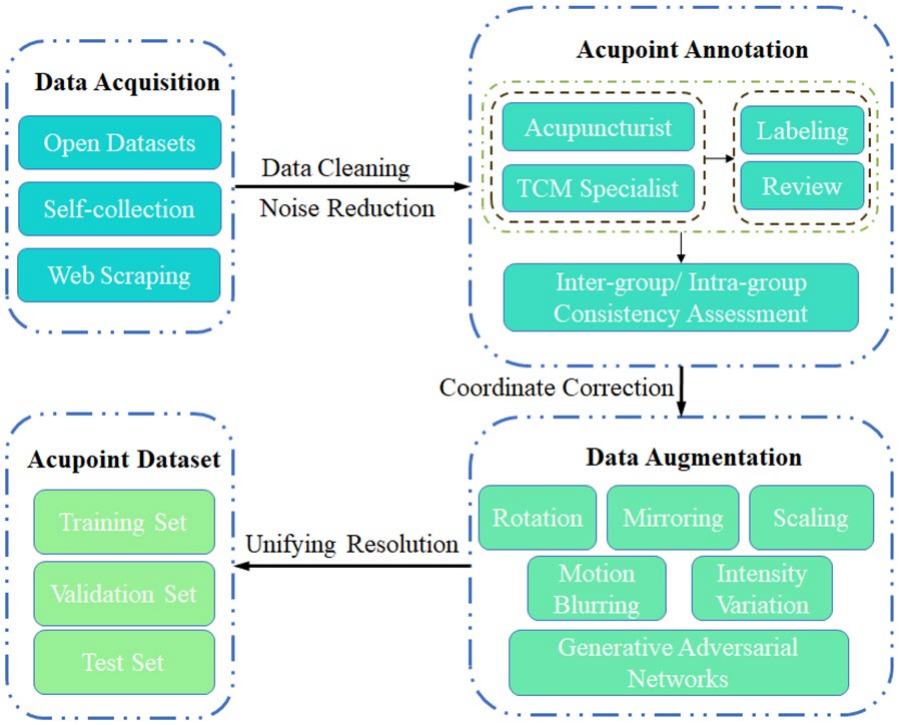
The construction process of the standard acupoint location dataset

Regarding the standard dataset-building process (Fig. 3), data collection comes first, through methods like selecting from public datasets, self-made device collection, and web crawling. Then, acupoint annotation is required. Considering that it relies on expert experience to a certain extent and there are differences in the experience of different experts, to ensure the reliability of annotation, some literatures have adopted the following measures: inviting multiple experts or providing professional training for multiple annotators so that they can complete acupoint calibration on the same dataset according to uniform standards. At the same time, inter-group and intra-group consistency evaluations are carried out on the annotated acupoint coordinates to ensure the accuracy and reliability of annotations. If the evaluation results show low consistency, the final ground-truth coordinates are determined by calculating the mean value. Finally, to enhance model performance, data augmentation is applied to the dataset, including rotation, mirroring, scaling, etc., or combining with GANs to increase the dataset size, optimizing the overall dataset quality.

Considering the significance of datasets for the accuracy and generalization of localization models, future efforts should focus on building larger, more diverse direct localization datasets, covering a wider range of body parts and environments, while also creating multi-modal datasets to leverage the rich features from different imaging techniques, thereby enhancing localization accuracy and model generalization.

### Evaluation metrics for acupoint localization

In human acupoint localization, point-based acupoints account for the majority. Therefore, when evaluating acupoint localization, methods from keypoint detection can be applied, where the error distance between the predicted and ground truth coordinates is calculated. Additionally, since acupoints have a certain range, ideas from small-object detection can also be adopted, evaluating the overlap between the predicted region and the true region. However, due to the lack of a unified evaluation standard, different researchers have designed various evaluation metrics. In this paper, we select the most frequently used and expressive metrics to provide a clearer and more reliable reference for acupoint localization evaluation.

#### Normalized mean error (NME)

NME normalizes the average coordinate error by the fixed distance between two points (usually the two most accurately located and distant points in the image). For example, in facial acupoint localization, the formula is:

1 $$NME = \frac{{\left( {1/n} \right)\mathop \sum \nolimits_{i = 1}^{n} \left\| {x_{i} - \hat{x}_{i} } \right\|_{2} }}{d}$$

where $n$ represents the number of acupoints, ${x}_{i}$ is the predicted coordinate of the $i$-th acupoint, ${\widehat{x}}_{i}$ is the ground truth coordinate, and $\parallel {x}_{i}-{\widehat{x}}_{i}{\parallel }_{2}$ represents the L2 norm (Euclidean distance) between the predicted and true coordinates. The normalization factor $d$ is typically the distance between the two outer corners of the eyes in facial acupoint localization. A more accurate regression of coordinates results in a lower NME.

#### Percentage of correct keypoints (PCK)

The PCK metric is used to assess the degree of alignment between detected keypoint positions and true positions in keypoint detection tasks. It is defined as the percentage of correctly detected keypoints, calculated as:

2 $$PCK = \frac{{\mathop \sum \nolimits_{i = 1}^{N} \delta \left( {d_{pi} \le \tau } \right)}}{N}$$

where $N$ is the total number of acupoints, ${d}_{pi}$ is ane Euclidean distance between the predicted and true coordinates for the $i$-th acupoint, $\tau$ is the threshold for normalized distance, and $\delta$ is an indicator function. If ${d}_{pi}\le \tau ,\delta \left({d}_{pi}\le \tau \right)=1;$ otherwise, $\delta \left({d}_{pi}\le \tau \right)=0.$ A higher PCK indicates more correct keypoints.

#### Keypoint similarity (OKS)

The OKS metric considers the Euclidean distance between keypoints, body scale, and keypoint weight. It is a widely used evaluation metric in keypoint detection tasks, calculated as:

3 $$OKS_{p} = \frac{{\mathop \sum \nolimits_{i = 1}^{N} e^{{ - \frac{{d_{pi}^{2} }}{{2s_{p}^{2} \sigma_{i}^{2} }}}} \delta \left( {v_{i} > 0} \right)}}{{\mathop \sum \nolimits_{i = 1}^{N} \delta \left( {v_{i} > 0} \right)}}$$

where $p$ denotes the detected person, $N$ is the total number of acupoints, ${d}_{pi}^{2}$ is the squared Euclidean distance between the predicted and true coordinates for the $i$-th acupoint, ${s}_{p}$ is the scale factor of the $p$-th person’s acupoint region, ${\sigma }_{i}$ is the normalization factor for the $i$-th acupoint, indicating the difficulty of detecting this key point, and ${v}_{i}$ is the visibility flag for the keypoint. If the keypoint is visible or occluded, ${v}_{i}>$ 0; otherwise, ${v}_{i}=0.$ OKS values range from 0 to 1, with a value of 1 indicating perfect alignment.

#### Mean average precision (mAP)

Similar to mAP used in object detection, mAP in acupoint localization considers both precision and recall, using OKS or Intersection over Union (loU) to measure the similarity between predicted and true values. The formula for mAP using OKS is as follows: first, rank predictions by confidence, then calculate precision and recall based on confidence $C$ and the OKS threshold $\theta$, where $T{P}_{\left(C|\theta \right)}$ represents true positives, $F{P}_{\left(C|\theta \right)}$ represents false positives, and $F{N}_{\uptheta }$ represents false negatives:

4 $$Precision_{{\left( {C|\theta } \right)}} = \frac{{TP_{{\left( {C|\theta } \right)}} }}{{TP_{{\left( {C|\theta } \right)}} + FP_{{\left( {C|\theta } \right)}} }}$$

5 $$Recall_{{\left( {C|\theta } \right)}} = \frac{{TP_{{\left( {C|\theta } \right)}} }}{{TP_{{\left( {C|\theta } \right)}} + FN_{{\left( {C|\theta } \right)}} }}$$

Then calculate the precision and recall at each confidence level, draw the precision-recall curve, and the area under the curve is AP, which can also be calculated using a formula:

6 $$AP = \mathop \smallint \limits_{0}^{1} Precision_{C\left| \theta \right.} dRecall_{C\left| \theta \right.}$$

Finally, average the APs of the N acupuncture points to get the mAP:

7 $$mAP = \frac{{\mathop \sum \nolimits_{i = 1}^{N} AP_{i} }}{N}$$

The higher the mAP, the better the model’s performance in acupoint localization. mAP commonly calculated at different thresholds, such as mAP@0.5, reflecting performance with more relaxed matching, and mAP@0.5:0.95, which evaluates the model’s performance under varying constraints.

#### Failure rate (FR)

FR is the proportion of acupoints with a localization error exceeding a set threshold. It is calculated as:

8 $$FR = \frac{{\mathop \sum \nolimits_{i = 1}^{N} \delta \left( {PM_{i} \ge \tau } \right)}}{N}$$

where $P{M}_{i}$ represents a specific localization accuracy metric for the $i$-th acupoint (e.g., Euclidean distance, NME, or OKS), and $\delta \left(P{M}_{i}\ge \tau \right)$ equals 1 if the accuracy metric exceeds the threshold $\tau$,or 0 otherwise.

#### Summary

The common evaluation metrics for acupoint localization, such as NME, OKS, PCK, FR, and mAP, are mostly derived from keypoint detection tasks. NME and OKS reflect the distance of localization error, while PCK and FR reflect the proportion of accurate localization. mAP offers a more comprehensive view of overall localization performance. Although these metrics are scientifically sound and accurate, most researchers still tend to use their own custom-designed metrics. A wider adoption of standardized metrics will facilitate better comparisons and comprehensive evaluations across studies.

Since acupoints are not merely precise points on the body surface but rather regions with specific sizes, their specific morphology (including size and shape) is influenced by various factors such as their location, acupuncture manipulation techniques, patient body type, disease characteristics, and severity. They can also exhibit non-circular irregular shapes. Considering the above factors and the differences among different experts or localization standards, we can model acupoint localization as a representative point (its position) plus a 2D Gaussian distribution around this point. The core region of this distribution (near the peak) yields the best or most specific therapeutic effect, while the effect diminishes as it deviates from the center. For example, some literatures divide the error range into intervals (such as 5 mm and 10 mm from the center) and calculate the localization accuracy within each interval respectively. This model-based understanding helps in personalized design and evaluation according to the characteristics of different acupoints. It also aids in avoiding overly absolute localization standards in clinical practice, thus better adapting to real-world scenarios.

## Application scenarios

Deep learning-driven automated acupoint localization technology, with its powerful data processing capabilities and intelligent algorithms, has opened new development pathways in clinical treatment, self-care, TCM education, diagnostic evaluation, and medical robotics, creating multifaceted opportunities.

### Assisted localization

In clinical TCM practices such as acupuncture, Tuina, and therapeutic massage, automated acupoint localization systems assist practitioners in rapidly and accurately identifying acupoints, thereby enhancing treatment efficacy and efficiency. These systems prove particularly valuable for inexperienced practitioners or non-professionals by providing robust auxiliary support, mitigating issues like suboptimal therapeutic outcomes or adverse reactions caused by localization inaccuracies. Furthermore, certain systems integrated with knowledge bases or large language models can offer personalized treatment recommendations based on patients’ constitutional characteristics and disease patterns, significantly improving the efficiency of TCM diagnosis and treatment.

### Self-care

With growing health consciousness, an increasing number of individuals are adopting TCM techniques like moxibustion, guasha, and massage for personal or familial healthcare. Automated acupoint localization applications empower the general public with convenient acupoint identification tools to conduct proper self-care. For instance, through mobile applications, users can retrieve disease-specific acupoints and follow guided instructions on massage techniques, hot/cold compress durations, and moxibustion protocols to perform relatively accurate healthcare procedures. Such applications not only enhance the convenience and effectiveness of self-care but also actively contribute to preventive healthcare strategies.

### TCM education

In TCM pedagogy, automated acupoint localization systems serve as didactic aids, enabling students to intuitively comprehend and master acupoint positions and characteristics, thereby improving learning outcomes. Educators can leverage automatically localized acupoint images and related information to elucidate meridian theory more effectively while strengthening students'practical skills. Augmented reality (AR) integration further facilitates immersive learning experiences through visualized acupoint localization, which not only preserves traditional knowledge but also stimulates student engagement and motivation.

### Diagnostic evaluation

Automated acupoint localization integrated with infrared thermography holds critical significance in TCM diagnostics. By directly measuring acupoint temperatures on thermal images and analyzing their correlations with pathological conditions, this technology enables effective detection of existing diseases and prediction of potential health risks. Furthermore, comparative analysis of pre- and post-treatment temperature variations provides objective quantitative metrics for therapeutic efficacy assessment. Additionally, temperature differentials across acupoints allow evaluation of qi-blood circulation states, offering comprehensive references for TCM diagnosis and treatment planning.

### TCM robotics

The intelligent acupuncture/moxibustion/massage robot integrates high-precision sensors, positioning algorithms, and mechanical structure design. It achieves precise acupoint positioning through a mechanical arm and simulates TCM techniques based on a massive database. Its intelligent functions cover prescription generation, treatment plan customization, precise regulation of moxibustion temperature, and safety monitoring, enabling personalized therapy. However, current technologies have obvious limitations: existing systems mostly target flat and easily accessible areas such as the back (typically requiring patients to assume a prone position), controlling acupuncture depth via stepper motors, adjusting massage intensity with pressure sensors, and positioning moxibustion sites using Z-axis coordinates. This technical model faces bottlenecks in clinical application, urgently requiring the development of multi-posture adaptive technologies and precise operation systems for complex areas. Especially in acupuncture, it is necessary to achieve depth-intensity coordinated control to meet the therapeutic needs of different tissues.

## Conclusions

This paper explores deep learning-based acupoint localization technology, analyzing and summarizing it from multiple perspectives. The research on facial and limb acupoints is relatively abundant due to lower privacy and easier dataset access. The methods are primarily divided into keypoint detection-based and GAN-based, with keypoint detection methods being more widely applied. Localization strategies are divided into speed-oriented and accuracy-oriented to meet diverse application needs. In terms of image modalities, depth images are prominent due to their use in TCM robotics, while thermal imaging and medical imaging research are limited. Datasets for direct acupoint annotation are mostly not open-source and limited, while indirect datasets from keypoint and object detection are more available. The application layer focuses on auxiliary localization, self-care, TCM education, diagnostic, evaluation and TCM robots, with auxiliary localization and TCM robots showing significant results. However, diagnostic evaluation lags due to high precision requirements.

Although progress has been made, several issues need addressing and optimization: dataset construction should follow the processes summarized to create more comprehensive datasets; model architecture should explore innovative approaches beyond CNN; localization strategies should balance speed and accuracy; evaluation metrics should be more targeted considering acupoint characteristics; the integration of image modalities and features from non-image data such as pressure sensing data, electrical resistance potential, etc., should be pursued to apply to more complex medical research scenarios; and integration with foundation models should be pursued to enhance intelligence and precision.

## Data Availability

No datasets were generated or analysed during the current study.

## Abbreviations

TCM: Traditional Chinese Medicine
DL: Deep learning
CNN: Convolutional neural network
RNN: Recurrent neural network
AE: Autoencoder
GAN: Generative adversarial network
ResNet: Residual network
MTCNN: Multi task cascaded convolutional networks
PFLD: Practical facial landmark detector
FC: Fully connected
NME: Normalized mean error
mAP: Mean average precision
AR: Augmented reality
ECA: Efficient channel attention
SSD: Single Shot MultiBox Detector
MOE: Mean offset error
FPS: Frames per second
SSTN: Symmetric spatial transformer network
CBAM: Convolutional block attention module
RCSAF: Resolution channel and spatial attention fusion
AUC: Area UNDER THE CURVE
MLP: Multilayer perceptron
PCK: Percentage of correct keypoints
OKS: Object keypoint similarity
MAE: Mean absolute error
RMSE: Root mean squared error
FR: Failure rate
RGB-D: Red green blue depth
CT: Computed tomography
SMPL: Skinned multi person linear
SSIM: Structural similarity index measure
ICC: Intraclass correlation coefficient
IoU: Intersection over union
RPN: Region Proposal Network
TP: True positive
FP: False positive
FN: False negative
AP: Average precision
3DMM: 3D morphable model
PAFs: Part affinity fields
RMPE: Regional multi person pose estimation
CPN: Cascaded pyramid network
CPM: Convolutional pose machine
DCGAN: Deep convolutional generative adversarial network
UV: Texture coordinates
XYZ: Cartesian coordinates
WHO: World Health Organization
GB/T: Guobiao Standard (Chinese National Standard)
